# Remote Patient Monitoring in Heart Failure: A Systematic Review, Meta-Analysis, and Trial Sequential Analysis

**DOI:** 10.7759/cureus.109198

**Published:** 2026-05-19

**Authors:** Vicky Muller Ferreira, Victor Ayres Muller

**Affiliations:** 1 Cardiology, Independent Researcher, Rio de Janeiro, BRA; 2 Internal Medicine/Clinical Research, Hospital Universitário de Vassouras, Rio de Janeiro, BRA

**Keywords:** cardiomems, grade, heart failure, meta-analysis, remote patient monitoring, structured telephone support, telemonitoring, trial sequential analysis

## Abstract

Whether the cumulative evidence for remote patient monitoring (RPM) in heart failure (HF) is robust to sequential monitoring, and whether trials report geographic access modifiers, remains uncertain. We conducted a systematic review, meta-analysis, and trial sequential analysis (TSA) of 65 randomized controlled trials (RCTs) (59 poolable; approximately 23,000 participants) identified through a search of PubMed/MEDLINE, Cochrane CENTRAL, ClinicalTrials.gov, and the WHO International Clinical Trials Registry Platform (ICTRP) from inception through February 15, 2026, encompassing structured telephone support (STS) (15 trials), non-invasive telemonitoring (TM) (33 trials), and invasive hemodynamic monitoring (11 trials). Random-effects meta-analysis used restricted maximum likelihood (REML) with the Hartung-Knapp-Sidik-Jonkman (HKSJ) adjustment, and certainty of evidence was rated using the Grading of Recommendations, Assessment, Development, and Evaluations (GRADE) framework. RPM significantly reduced all-cause mortality (ACM) (risk ratio (RR): 0.911, 95% confidence interval (CI): 0.842-0.985; P=0.021; I²=0%; k=41; number needed to treat (NNT) 104 per year; prediction interval: 0.840-0.988), and trial sequential analysis suggested that the accrued evidence exceeded the required information size under a 15% relative risk reduction assumption, supporting a stable mortality signal. HF hospitalization was reduced (RR: 0.781, 95% CI: 0.710-0.859; P<0.001; k=39; number needed to treat 18 per year), although the prediction interval crossed 1.0 (0.586-1.040), indicating that in some clinical settings, the effect may be attenuated. All-cause hospitalization was not significantly reduced (RR: 0.959, 95% CI: 0.892-1.031; k=28). No statistically significant interaction by RPM modality was detected for any primary outcome (all-cause mortality P-interaction=0.80; HF hospitalization P-interaction=0.14). GRADE certainty was moderate for all-cause mortality and low for HF hospitalization, downgraded primarily for suspected publication bias and inconsistency. A descriptive geographic access analysis revealed that only 2 of 59 poolable trials reported formal rural-versus-urban subgroup analyses, precluding firm conclusions about whether RPM differentially benefits geographically underserved populations. Within these limitations, remote patient monitoring appears to reduce all-cause mortality and HF hospitalization compared with usual care across diverse modalities, while signaling persistent gaps in heterogeneity reporting and equity-focused subgroup data.

## Introduction and background

Heart failure (HF) affects over 64 million people worldwide and remains a leading cause of hospitalization, disability, and death [1]. Despite advances in pharmacological therapy, HF hospitalization rates remain high, with 30-day readmission rates exceeding 20% in many health systems [2]. The burden is disproportionately borne by patients in rural and underserved areas, where access to specialized HF care is limited [3].

Remote patient monitoring (RPM) encompasses a spectrum of technologies designed to extend clinical oversight beyond the hospital or clinic setting. Three broad categories have been studied in HF: structured telephone support (STS), in which nurses or automated systems contact patients at regular intervals to assess symptoms and guide self-management; non-invasive telemonitoring (TM), which involves the electronic transmission of physiological data (weight, blood pressure, heart rate, and oxygen saturation) from the patient's home to a clinical team; and invasive hemodynamic monitoring, using implanted sensors (such as the CardioMEMS pulmonary artery pressure sensor) or cardiac implantable electronic device (CIED) algorithms to detect congestion before clinical decompensation.

The evidence base for RPM in HF has grown substantially over the past two decades. The 2015 Cochrane review by Inglis et al. established that both STS and non-invasive TM reduced all-cause mortality (ACM) compared with usual care, with mortality risk ratios (RR) of 0.87 for STS and 0.80 for non-invasive TM [4]. Since then, several landmark trials have been published. The TIM-HF2 trial demonstrated that structured remote management reduced the percentage of days lost to unplanned cardiovascular (CV) hospitalization or death [5], while the CHAMPION and MONITOR-HF trials showed that hemodynamic-guided management with the CardioMEMS sensor reduced HF hospitalizations [6,7]. The GUIDE-HF trial, however, yielded neutral overall results, with benefit confined to a pre-COVID sensitivity analysis [8]. The recently published PROACTIVE-HF trial expanded invasive monitoring to HF with preserved ejection fraction (HFpEF) [9], and the MIGHTY-HEART trial tested a smartphone-based coaching intervention in a diverse urban population, although its primary composite endpoint was neutral [10].

Despite this growing evidence, important questions remain unanswered. First, no prior meta-analysis has applied trial sequential analysis (TSA) to evaluate whether the cumulative evidence for RPM is robust to repeated testing as trials accrue. Second, the most recent comprehensive meta-analysis predates several important trials (PROACTIVE-HF, MIGHTY-HEART, PRADOC, MESSAGE-HF, and MONITOR-HF). Third, whether the benefits of RPM extend to geographically underserved populations, where RPM could theoretically provide the greatest incremental value, has not been systematically examined, despite evidence from TIM-HF2 suggesting that patients farther from cardiologists derive greater benefit [5]. Fourth, HF phenotype-specific data (HF with reduced ejection fraction (HFrEF), HF with preserved ejection fraction (HFpEF), and HF with mildly reduced ejection fraction (HFmrEF)) remain limited, as most trials enrolled mixed populations.

We therefore conducted a systematic review, meta-analysis, and TSA to evaluate the effects of RPM on mortality, hospitalization, and patient-reported outcomes in adults with HF, with the primary objectives of (1) assessing whether the cumulative evidence is robust to sequential monitoring and (2) describing geographic access reporting and exploratory effect modification.

## Review

Methodology

Protocol and Registration

This systematic review was registered with PROSPERO (CRD420261323628) and conducted in accordance with the Preferred Reporting Items for Systematic Reviews and Meta-Analyses (PRISMA) 2020 guidelines [11]. Searches were completed before registration; this timing and subsequent protocol amendments are documented in the online data repository (DOI: 10.5281/zenodo.19865261). The completed PRISMA 2020 checklist is available in the same repository.

Eligibility Criteria

We included randomized controlled trials (RCTs) that enrolled adults with heart failure of any left ventricular ejection fraction (LVEF) phenotype, randomized to any form of remote patient monitoring versus usual care or standard care, with a minimum follow-up of three months. Detailed inclusion and exclusion criteria, organized by population, intervention, comparator, outcomes, study design, sample size, follow-up, and publication type, are presented in Table 1.

**Table 1:** Eligibility criteria Inclusion and exclusion criteria for trial selection, with each criterion presented in a separate row and organized by domain (Population, Intervention, Comparator, Outcomes, Study design, Sample size, Follow-up, and Publication type). HF: heart failure, LVEF: left ventricular ejection fraction, HFrEF: heart failure with reduced ejection fraction, HFmrEF: heart failure with mildly reduced ejection fraction, HFpEF: heart failure with preserved ejection fraction, NYHA: New York Heart Association, RPM: remote patient monitoring, BP: blood pressure, HR: heart rate, ICD: implantable cardioverter-defibrillator, CRT-D: cardiac resynchronization therapy defibrillator, MLHFQ: Minnesota Living With Heart Failure Questionnaire, KCCQ: Kansas City Cardiomyopathy Questionnaire

Domain	Inclusion	Exclusion
Population	Adults aged ≥18 years with an established diagnosis of heart failure of any LVEF phenotype (HFrEF ≤40%, HFmrEF 41%-49%, or HFpEF ≥50%) and any NYHA functional class	Pediatric or adolescent populations; non-HF cardiovascular disease as primary diagnosis
Intervention	Any form of RPM, including structured telephone support, non-invasive telemonitoring (e.g., weight, BP, HR, oxygen saturation, mHealth, and wearables), or invasive hemodynamic monitoring (e.g., CardioMEMS and ICD/CRT-D-based device monitoring)	RPM intervention bundled with components that cannot be isolated (e.g., cardiac rehabilitation programs)
Comparator	Usual care or standard care (no RPM)	Active comparator that is also an RPM technology
Outcomes	At least one outcome of interest reported: all-cause mortality, HF hospitalization, all-cause hospitalization, cardiovascular mortality, composite outcomes, emergency department visits, or quality of life (e.g., MLHFQ and KCCQ)	Studies reporting only surrogate or process outcomes
Study design	Randomized controlled trials (parallel-group, cluster, factorial, or crossover with extractable first-period data)	Observational studies; single-arm trials; before-and-after designs; non-randomized comparative studies
Sample size	≥20 participants	Studies with fewer than 20 participants
Follow-up	Minimum follow-up of 3 months	Follow-up <3 months
Publication type	Peer-reviewed full publications in any language	Conference abstracts without an associated full publication; protocol papers without primary results

Information Sources and Search Strategy

We searched PubMed/MEDLINE, Cochrane CENTRAL, ClinicalTrials.gov, and the WHO International Clinical Trials Registry Platform (ICTRP) from inception through February 15, 2026, without language restrictions. The search strategy combined Medical Subject Headings (MeSH) and free-text terms for remote monitoring technologies (including telemonitoring, telehealth, structured telephone support, CardioMEMS, home monitoring, mHealth, and wearable devices) and heart failure, with an RCT filter. The full electronic search strategies for all four databases, including Boolean syntax, field tags, and per-database record yields, are presented in Table 2. The protocol was registered with English-language restriction; however, the search was subsequently conducted without language restriction to maximize sensitivity (protocol amendment #6). Embase and CINAHL were not searched because institutional access was unavailable. To partially mitigate this limitation, we relied on Cochrane CENTRAL, which aggregates records from multiple sources, including Embase: 42.9% of the 2,138 CENTRAL records retrieved in our search carried an Embase identifier. PubMed combined with CENTRAL captures approximately 97% of relevant RCTs in therapeutic systematic reviews [12,13]. A post-hoc cross-referencing analysis against three contemporary meta-analyses that searched additional databases [14-16] was conducted to assess the potential impact of this omission (Appendix A).

**Table 2:** Database search strategies Search strings used to query each bibliographic database, with the number of records retrieved per database, and a date column indicating when the search was executed. ICTRP: International Clinical Trials Registry Platform

Database	Search strategy	Records retrieved	Date
PubMed/MEDLINE	(("telemedicine"[MeSH] OR "remote consultation"[MeSH] OR "monitoring, physiologic"[MeSH] OR "telemonitor*"[tiab] OR "tele-monitor*"[tiab] OR "remote monitor*"[tiab] OR "telehealth"[tiab] OR "telemedicine"[tiab] OR "mHealth"[tiab] OR "mobile health"[tiab] OR "structured telephone support"[tiab] OR "telephone follow*"[tiab] OR "phone follow*"[tiab] OR "home monitor*"[tiab] OR "home telecare"[tiab] OR "digital health"[tiab] OR "implantable hemodynamic"[tiab] OR "CardioMEMS"[tiab] OR "pulmonary artery pressure monitor*"[tiab] OR "wearable*"[tiab] OR "smartphone*"[tiab] OR "app-based"[tiab]) AND ("heart failure"[MeSH] OR "heart failure"[tiab] OR "cardiac failure"[tiab] OR "HFrEF"[tiab] OR "HFpEF"[tiab] OR "cardiomyopathy"[tiab]) AND ("randomized controlled trial"[pt] OR "controlled clinical trial"[pt] OR "randomized"[tiab])) NOT (animals[mh] NOT humans[mh])	1,387	February 15, 2026
Cochrane CENTRAL	#1 [mh "Telemedicine"]; #2 [mh "Remote Consultation"]; #3 [mh "Monitoring, Physiologic"]; #4 (telemonitor* OR tele-monitor* OR (remote NEXT monitor*) OR telehealth OR telemedicine):ti,ab,kw; #5 (mHealth OR (mobile NEXT health) OR (structured NEXT telephone NEXT support) OR (telephone NEXT follow*)):ti,ab,kw; #6 ((home NEXT monitor*) OR (home NEXT telecare) OR (digital NEXT health)):ti,ab,kw; #7 (CardioMEMS OR (implantable NEXT hemodynamic) OR (pulmonary NEXT artery NEXT pressure NEXT monitor*)):ti,ab,kw; #8 (wearable* OR smartphone* OR app-based):ti,ab,kw; #9 #1 OR #2 OR #3 OR #4 OR #5 OR #6 OR #7 OR #8; #10 [mh "Heart Failure"]; #11 ((heart NEXT failure) OR HFrEF OR HFpEF OR cardiomyopathy):ti,ab,kw; #12 #10 OR #11; #13 #9 AND #12 (Trials filter applied)	2,138	February 15, 2026
ClinicalTrials.gov	AREA[ConditionSearch] "Heart Failure" AND AREA[InterventionSearch] (telemonitoring OR telehealth OR "remote monitoring" OR "structured telephone support" OR "telephone follow-up" OR CardioMEMS OR "pulmonary artery pressure" OR "home monitoring" OR mHealth OR wearable OR smartphone OR "digital health") AND AREA[StudyType] INTERVENTIONAL	348	February 15, 2026
WHO ICTRP	Four parallel searches combined: (1) Title: telemonitoring AND heart failure | Condition: heart failure; (2) Title: telehealth AND heart failure | Condition: heart failure; (3) Title: remote monitoring AND heart failure | Condition: heart failure; (4) Title: CardioMEMS OR pulmonary artery pressure | Condition: heart failure. Recruitment status: ALL.	164	February 15, 2026
Total identified	Combined across 4 databases (before deduplication)	4,037	-

Study Selection

Records were imported, combined, and deduplicated using a multi-pass approach (exact DOI/PMID matching, normalized title matching, and fuzzy matching with manual adjudication). Title and abstract screening used a semi-automated approach with predefined exclusion rules validated against a reference set of known eligible trials. Records flagged by the algorithm (n=219, 8.3%) underwent manual adjudication with final consensus decisions retained in the screening dataset. A post-hoc sensitivity audit of the screening algorithm identified two false negatives due to missing search terms (telemanagement and congestive heart failure (CHF)), which were rescued and included. Records passing initial screening underwent a structured audit to exclude registry-only entries, protocols, conference abstracts, and non-RCT designs. All remaining records were assessed at the full-text level using structured eligibility tiers. The archived screening datasets preserve final consensus decisions rather than complete independent reviewer-level decisions; therefore, the process should be interpreted as semi-automated screening with manual adjudication and audit rather than fully independent dual screening. Detailed algorithm documentation and the post-hoc audit log are provided in Appendix B.

Data Extraction

Data were extracted using a two-reviewer process. One reviewer (VMF) performed the initial structured extraction with AI-assisted tabulation support, and the second reviewer (VAM) independently reviewed the extracted data against source reports; discrepancies were resolved by consensus before analysis. The archived extraction dataset reports the final consensus values used for analysis; reviewer-level intermediate disagreement fields were not retained for every study. Quality checks identified and corrected three errors during the consensus process: MONITOR-HF HF hospitalization HR (0.82 corrected to 0.56, verified against original publication Table 2), CHAMPION PMID, and the CO-0056 mortality denominator (corrected from the pooled intervention denominator to the telephone arm denominator). Extracted effect estimates were checked against original publication tables and supplementary materials where available; extraction notes and a consensus log documenting reviewer roles, correction flags, and final verification status for all 65 studies are available in the Zenodo repository. Data were extracted using a standardized form encompassing 185 variables, including study characteristics, participant demographics, intervention details, geographic context (rural/urban classification and distance to care), outcomes (event counts for binary outcomes; means and standard deviations for continuous outcomes; hazard ratios with 95% confidence intervals (CI) when reported), and risk of bias domains. RPM interventions were classified into three prespecified categories: structured telephone support (STS), non-invasive telemonitoring, and invasive hemodynamic monitoring.

Risk of Bias Assessment

Risk of bias was assessed using the Cochrane Risk of Bias 2 (RoB 2) tool [17] across five domains: randomization process (D1), deviations from intended interventions (D2), missing outcome data (D3), measurement of the outcome (D4), and selection of the reported result (D5). All RPM trials are inherently open-label for participants and clinical staff; therefore, D2 ratings reflect whether knowledge of group assignment could have influenced co-interventions or adherence beyond the intended effect.

Effect Measures and Statistical Analysis

The primary outcomes were all-cause mortality, HF hospitalization, and all-cause hospitalization. Secondary outcomes included cardiovascular mortality, composite of all-cause mortality and hospitalization, emergency department visits, and quality of life (Minnesota Living with Heart Failure Questionnaire (MLHFQ) and Kansas City Cardiomyopathy Questionnaire (KCCQ)).

For binary outcomes with event counts, risk ratios (RR) were calculated using the Mantel-Haenszel method. Rows in which event counts represented recurrent events or episodes rather than participants with events (events exceeding denominators) were excluded from binary RR pooling and from control event rate (CER)/number needed to treat (NNT) calculations. Studies reporting only hazard ratios (HR) with 95% confidence intervals (CI), without extractable event counts, were not included in the primary RR analysis but were pooled separately using the generic inverse-variance method with log-transformed HRs and corresponding standard errors. This yielded two parallel analyses for each primary outcome: a primary RR analysis maximizing the number of contributing studies with valid participant-level event counts (e.g., k=41 for all-cause mortality) and a sensitivity HR-only analysis restricted to time-to-event data (e.g., k=16 for all-cause mortality). Continuous outcomes were pooled as mean differences (MD). RR and HR estimates are reported separately throughout and are not combined in a single model, as mixing effect measures can introduce bias [18]. For trials reporting outcomes at multiple time points, events at the protocol-specified primary endpoint were extracted (e.g., CHAMPION at 6 months rather than extended follow-up at 15-18 months). The cluster-randomized trial (CHAT) was adjusted using the design effect (DE) method (DE = 1.35, based on intraclass correlation coefficient (ICC) = 0.02 and mean cluster size 18.4), with both event counts and denominators reduced per Cochrane Handbook Section 23.1.3 [19].

Random-effects meta-analysis was performed using restricted maximum likelihood (REML) estimation for the between-study variance (τ²) with the Hartung-Knapp-Sidik-Jonkman (HKSJ) adjustment for confidence intervals [20,21]. Statistical heterogeneity was assessed using I², τ², Cochran's Q test, and 95% prediction intervals [22,23].

Subgroup and Sensitivity Analyses

Prespecified subgroup analyses examined the influence of RPM type (STS, non-invasive TM, and invasive), HF phenotype (HF with reduced ejection fraction (HFrEF), HF with preserved ejection fraction (HFpEF), HF with mildly reduced ejection fraction (HFmrEF), and mixed), geographic context (rural/remote, urban, mixed, and not reported), country income level (high-income versus other), follow-up duration (≤6 months versus >6 months), publication era (≤2015 versus >2015), and risk of bias (high versus low/some concerns). Interaction P-values were calculated using the Q-test for between-subgroup differences.

Ten prespecified sensitivity analyses were performed for each primary outcome: (1) leave-one-out analysis, (2) fixed-effect (common-effect) model, (3) exclusion of active comparator arms, (4) exclusion of invasive RPM studies, (5) exclusion of cluster-randomized trials, (6) exclusion of high risk of bias studies, (7) exclusion of flagged outliers, (8) restriction to HR-reporting studies only, (9) restriction to follow-up ≥3 months, and (10) restriction to large trials (N≥200). Meta-regression examined the association of treatment effect with total sample size, publication year, follow-up duration, mean age, and mean LVEF.

Publication Bias

Publication bias was assessed visually using funnel plots and statistically using Egger's regression test [24], Begg's rank correlation test, and Peters' regression test. The trim-and-fill method was applied to estimate the impact of potential missing studies [25]. Contour-enhanced funnel plots were generated to distinguish asymmetry due to publication bias from asymmetry due to other causes. A large-trial sensitivity analysis (N≥200) was performed to assess whether small-study effects drove the overall signal.

Trial Sequential Analysis

Trial sequential analysis (TSA) was performed using α=0.05, β=0.20 (80% power), and relative risk reductions (RRR) of 15% and 20%, with O'Brien-Fleming spending boundaries [26]. TSA was used as a sensitivity framework to assess whether cumulative evidence was robust to repeated testing under the specified assumptions.

Certainty of Evidence

The certainty of evidence was assessed using the Grading of Recommendations, Assessment, Development, and Evaluations (GRADE) framework [27] across five domains: risk of bias, inconsistency, indirectness, imprecision, and publication bias.

All analyses were performed in R (version 4.5.2) using the meta [28] and metafor [29] packages. Statistical significance was defined as P<0.05 (two-sided).

Findings

Study Selection

**Figure 1:**
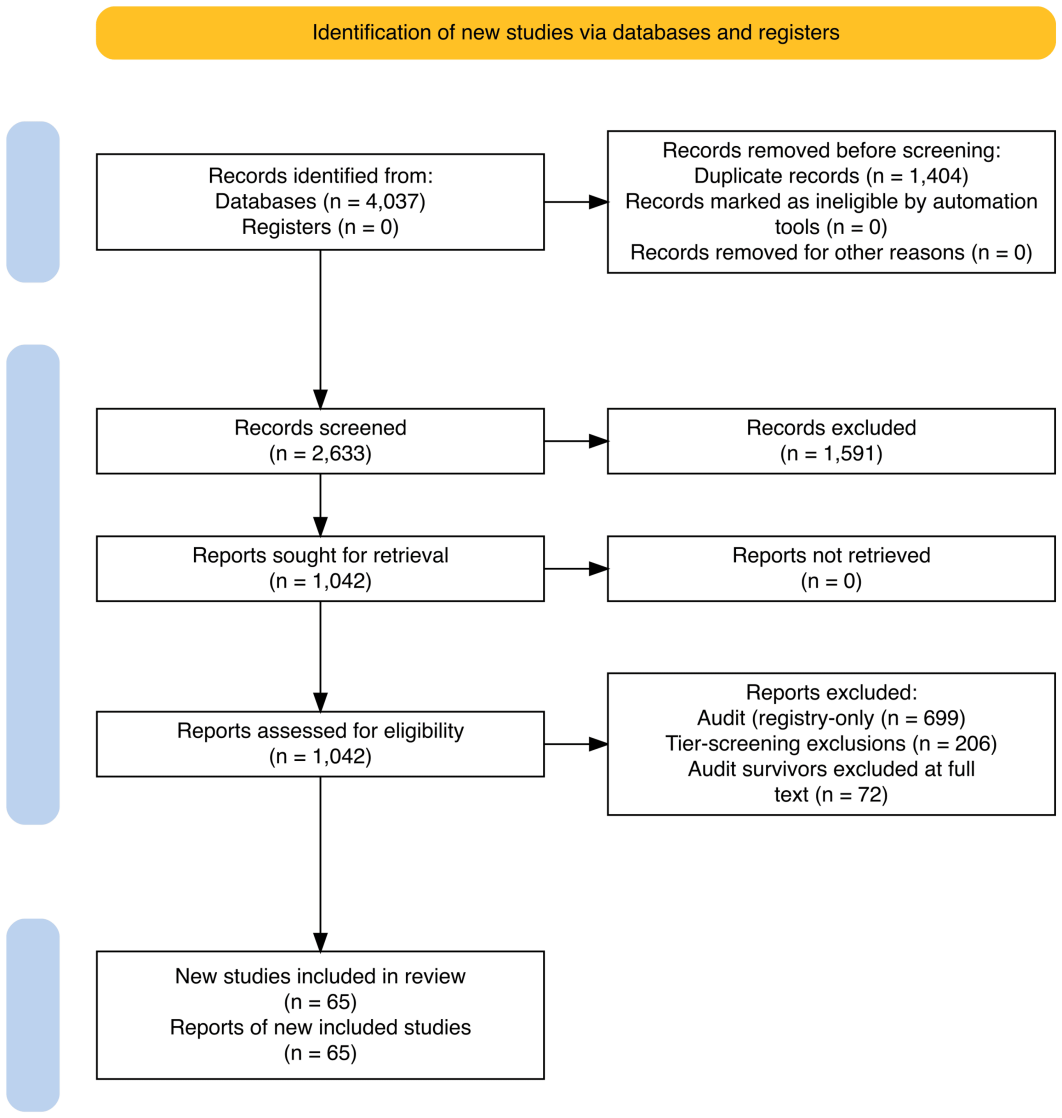
PRISMA 2020 flow diagram of study selection A total of 4,037 records were identified across four databases, and after deduplication, screening, and full-text assessment, 65 studies were included, of which 59 were poolable for quantitative synthesis. PRISMA: Preferred Reporting Items for Systematic Reviews and Meta-Analyses

The systematic search identified 4,037 records across four databases: PubMed (n=1,387), Cochrane CENTRAL (n=2,138), ClinicalTrials.gov (n=348), and WHO ICTRP (n=164). After identifying 1,405 duplicate records and rescuing one that had been incorrectly flagged, 1,404 records were removed, yielding 2,633 unique records for title and abstract screening. Of these, 1,616 were excluded, and 1,017 records proceeded to full-text assessment. A structured audit excluded 701 records (registry entries, protocols, conference abstracts, and non-RCTs), while 26 records were added or reclassified into the full-text pool (tier B candidates, manual review survivors, and pipeline rescues), yielding 342 records for full-text review across four tiers (A-D). After a detailed eligibility evaluation, 65 studies met all inclusion criteria (including two records rescued from title/abstract screening via post-hoc algorithm sensitivity analysis), of which 59 were poolable for quantitative synthesis (Figure 1).

The 65 included RCTs [5-10,30-88] encompassed approximately 23,000 participants across 20 countries. RPM interventions were classified as structured telephone support (15 trials), non-invasive telemonitoring (33 trials), or invasive hemodynamic monitoring (11 trials), with 6 trials excluded from quantitative synthesis because they used active comparators, had single-arm or before/after designs, or reported only outcomes incompatible with pooling. The median sample size was 244 (range: 25-1,653), and the median follow-up was 6 months (range: 2-48 months; one study (Negarandeh et al. (2019) [52]) had a 2-month follow-up and was retained by investigator decision as a borderline case). HF phenotype was predominantly HFrEF or mixed; no trials enrolled patients with HFpEF exclusively among the 59 poolable studies. Detailed study and intervention characteristics are presented in Table 3 and Table 4.

**Table 3:** Characteristics of included studies Age shown as mean ± SD BIDMC: Beth Israel Deaconess Medical Center, CHF: congestive heart failure, FU: follow-up, HF: heart failure, Int: intervention, Ctrl: control, LVEF: left ventricular ejection fraction, NEMC: New England Medical Center, NR: not reported, NYHA: New York Heart Association, RIH: Rhode Island Hospital, RoB: risk of bias, RPM: remote patient monitoring, STS: structured telephone support, TM: telemonitoring, RCT: randomized controlled trial, HFrEF: heart failure with reduced ejection fraction, SD: standard deviation

Study	Reference	Trial name	RPM type	Design	N (Int)	N (Ctrl)	Follow-up (months)	Age	Female (%)	HF type	LVEF (%)	NYHA III-IV (%)	Setting	Country	RoB overall
CHAMPION (Abraham et al. (2011))	[6]	CHAMPION	Invasive	Parallel RCT	270	280	6	61.6±12.8	27	Mixed	23	100	NR	USA	Some concerns
REDUCEhf (Adamson et al. (2011))	[34]	REDUCEhf	Invasive	Parallel RCT	202	198	12	55.0±15.0	30	HFrEF	23±8	51	Outpatient	USA	Some concerns
EVOLVO (Landolina et al. (2012))	[33]	EVOLVO	Invasive	Parallel RCT	99	101	16	NR	18	HFrEF	NR	17	Outpatient	Italy	Some concerns
IN-TIME (Hindricks et al. (2014))	[30]	IN-TIME	Invasive	Parallel RCT	333	331	12	65.3±9.3	18	HFrEF	26±6	57	Tertiary hospital (36 centers, 7 countries: Australia, Europe, Israel)	Germany	Some concerns
CONNECT-OptiVol (Lüthje et al. (2015))	[38]	CONNECT-OptiVol	Invasive	Parallel RCT	87	89	15	66.0±12.0	20	HFrEF	33±11	43	Inpatient implant/outpatient follow-up (single-center, tertiary care)	Germany	High
OptiLink HF (Böhm et al. (2016))	[37]	OptiLink HF	Invasive	Parallel RCT	505	497	22.8	66.1±10.1	23	HFrEF	27±6	81	Outpatient	Germany	Some concerns
TELECART (Sardu et al. (2016))	[36]	TELECART	Invasive	Parallel RCT	96	95	12	71.8±8.5	28	HFrEF	NR	55	Multicenter (3 Italian centers); outpatient + device clinic	Italy	Some concerns
MORE-CARE (Boriani et al. (2017))	[31]	MORE-CARE	Invasive	Parallel RCT	437	428	24	66.0±11.0	21	HFrEF	27±7	63	Outpatient	Italy (multinational)	Some concerns
RESULT (Tajstra et al. (2020))	[32]	RESULT	Invasive	Parallel RCT	299	301	12	NR	18	HFrEF	NR	22	Outpatient	Poland	Some concerns
GUIDE-HF (Lindenfeld et al. (2021))	[8]	GUIDE-HF	Invasive	Parallel RCT	497	503	12	71.0	38	Mixed	38	71	Outpatient	USA/Canada	Some concerns
MONITOR-HF (Brugts et al. (2023))	[7]	MONITOR-HF	Invasive	Parallel RCT	176	172	48	66.9±11.4	22	Mixed	30	100	Outpatient (25 hospitals, Netherlands)	Netherlands	Some concerns
Jerant 2001 (3-arm) (Jerant et al. (2001))	[81]	Jerant 2001 (3-arm)	Non-invasive TM	Parallel RCT	13	12	6	NR	NR	NR	NR	NR	Post-discharge	USA	High
Benatar et al. (2003)	[87]	-	Non-invasive TM	Parallel RCT	108	108	3	62.9±13.2	64	Mixed	38	NR	Outpatient/post-discharge	USA	Some concerns
Jerant 2003 (3-arm) (Jerant et al. (2003))	[79]	Jerant 2003 (3-arm)	Non-invasive TM	3-arm RCT	13	12	6	NR	NR	NR	NR	NR	Post-discharge	USA	High
TEN-HMS (Cleland et al. (2005))	[85]	TEN-HMS	Non-invasive TM	3-arm RCT	173	85	8	67.0±13.0	20	HFrEF	25±8	31	Outpatient/post-discharge (patients recently discharged for HF)	Germany/Netherlands/UK	Some concerns
Antonicelli et al. (2008)	[76]	-	Non-invasive TM	Parallel RCT	28	29	12	77.0±8.0	43	Mixed	35±6	42	Post-discharge	Italy	Some concerns
Dansky 2008 (Dansky and Vasey (2008))	[74]	Dansky 2008	Non-invasive TM	Parallel RCT	172	112	4	77.1	NR	NR	NR	NR	Post-discharge	USA	High
Kashem 2008 (Kashem et al. (2008))	[75]	Kashem 2008	Non-invasive TM	Parallel RCT	24	24	12	53.0±10.0	28	HFrEF	25±3	NR	Outpatient	United States	Some concerns
Dansky 2009 (Health Buddy) (Dansky et al. (2009))	[68]	Dansky 2009 (Health Buddy)	Non-invasive TM	Parallel RCT	64	46	6	76.9	NR	NR	NR	NR	Post-discharge	USA	High
Home-HF (Dar et al. (2009))	[71]	Home-HF	Non-invasive TM	-	91	91	6	70.0±12.8	32	Mixed	NR	NR	Post-discharge	UK	Some concerns
Giordano et al. (2009)	[88]	0	Non-invasive TM	Parallel RCT	230	230	12	57.0±10.0	NR	NR	NR	NR	Outpatient	Italy	High
HHH (Mortara et al. (2009))	[70]	HHH	Non-invasive TM	-	301	160	12	60.0±12.0	14	HFrEF	28±7	NR	Home-based	UK (multinational)	High
MOBITEL (Scherr et al. (2009))	[72]	MOBITEL	Non-invasive TM	Parallel RCT	66	54	6	65.0	26	Mixed	25	87	Post-discharge	Austria	High
Tompkins 2010 (Tompkins and Orwat (2010))	[65]	Tompkins 2010	Non-invasive TM	Parallel RCT	193	197	6	76.7±8.2	42	NR	NR	NR	Outpatient/home	USA	Some concerns
TIM-HF (Koehler et al. (2011))	[63]	TIM-HF	Non-invasive TM	Parallel RCT	354	356	26	66.9±10.8	20	HFrEF	27±6	50	Outpatient ambulatory	Germany	Some concerns
THCM (Wade 2011) (Wade et al. (2011))	[62]	THCM (Wade 2011)	Non-invasive TM	Parallel RCT	164	152	6	75.8	58	NR	NR	NR	Post-discharge	USA	High
TEHAF (Boyne et al. (2012))	[61]	TEHAF	Non-invasive TM	Parallel RCT	197	185	12	71.0±11.9	42	Mixed	36	43	Outpatient HF clinic (3 centers: Maastricht, Heerlen, and Sittard)	Netherlands	Some concerns
TEMA-HF 1 (Dendale et al. (2012))	[86]	TEMA-HF 1	Non-invasive TM	Parallel RCT	80	80	6	75.9±9.6	38	Mixed	35±15	NR	Post-discharge	Belgium	Some concerns
Gámez-López 2012 (Gámez-López et al. (2012))	[59]	Gámez-López 2012	Non-invasive TM	4-arm RCT	52	52	11	71.0	NR	Mixed	NR	40	Post-discharge	Spain	Some concerns
Seto 2012 (Seto et al. (2012))	[60]	Seto 2012	Non-invasive TM	Parallel RCT	50	50	6	55.1±13.7	18	HFrEF	27±8	46	Outpatient heart function clinic	Canada	Some concerns
Delaney 2013 (Delaney et al. (2013))	[58]	Delaney 2013	Non-invasive TM	Parallel RCT	46	47	3	77.6	67	NR	NR	100	Post-discharge/home care	USA	Some concerns
Pedone 2015 (Pedone et al. (2015))	[55]	Pedone 2015	Non-invasive TM	Parallel RCT	50	46	6	79.9±6.8	53	Mixed	44±13	68	Mixed	Italy	High
TIM-HF2 (Koehler et al. (2018))	[5]	TIM-HF2	Non-invasive TM	Parallel RCT	765	773	12	70.0±11.0	30	Mixed	41±13	47	Outpatient (post-hospitalization)	Germany	Some concerns
Renewing Health (Olivari et al. (2018))	[54]	Renewing Health	Non-invasive TM	Parallel RCT	229	110	12	NR	NR	Mixed	NR	NR	Post-discharge	Italy (multinational)	Some concerns
Pekmezaris 2019 (Pekmezaris et al. (2019))	[53]	Pekmezaris 2019	Non-invasive TM	Parallel RCT	46	58	3	58.4±15.2	41	Mixed	NR	70	Post-discharge	USA	Some concerns
OSICAT (Galinier et al. (2020))	[51]	OSICAT	Non-invasive TM	Parallel RCT	482	455	18	70.0±12.4	27	Mixed	39±14	49	Outpatient/post-discharge (hospitalized for acute HF ≤12 months before inclusion)	France	Some concerns
Yanicelli 2021 (Yanicelli et al. (2021))	[49]	Yanicelli 2021	Non-invasive TM	Parallel RCT	20	20	3	NR	34	HFrEF	36±12	30	Outpatient	Argentina	High
Johnson 2022 (Johnson et al. (2022))	[48]	Johnson 2022	Non-invasive TM	Parallel RCT	16	15	3	60.4±12.9	38	Mixed	NR	NR	Inpatient → post-discharge (academic hospital)	USA	High
AMULET (Krzesiński et al. (2022))	[47]	AMULET	Non-invasive TM	Parallel RCT	300	305	12	67.0±16.0	21	Mixed	32±15	25	Outpatient ambulatory clinics (9 sites)	Poland	Some concerns
ControlVit (Achury-Saldaña et al. (2024))	[44]	ControlVit	Non-invasive TM	Parallel RCT	70	70	6	64.1±12.0	30	HFrEF	30±10	11	Outpatient	Colombia	Some concerns
Arnar 2025 (Arnar et al. (2025))	[43]	Arnar 2025	Non-invasive TM	Parallel RCT	86	89	12	64.7±13.2	34	Mixed	44±13	13	Outpatient HF clinic (Landspitali - The National University Hospital of Iceland, Reykjavik; Iceland's only specialized HF outpatient clinic) + Reykjavik Heart Center	Iceland	Some concerns
M-Cardio (ERICA-HF) (Rustambekova et al. (2025))	[41]	M-Cardio (ERICA-HF)	Non-invasive TM	-	127	117	12	60.8±7.2	37	HFmrEF	42±12	78	Post-discharge	Kyrgyzstan	Some concerns
Vietnam HFrEF RCT (Tran et al. (2025))	[40]	Vietnam HFrEF RCT	Non-invasive TM	Parallel RCT	87	83	6	60.9±15.3	26	HFrEF	33±6	37	Outpatient	Vietnam	Some concerns
TeleMedBot (Zheleznykh et al. (2025))	[39]	TeleMedBot	Non-invasive TM	Parallel RCT	57	43	6	61.5±13.1	26	Mixed	46±12	NR	Post-discharge	Russia	High
Riegel et al. (2002)	[80]	-	STS	Parallel RCT	130	228	6	72.5±13.1	46	Mixed	42±17	98	Post-discharge	United States	Some concerns
Laramee et al. (2003)	[78]	-	STS	Parallel RCT	141	146	3	70.6±11.4	42	Mixed	NR	38	Post-discharge	United States	Some concerns
DIAL (GESICA Investigators 2005)	[83]	DIAL	STS	Parallel RCT	760	758	24	64.8±13.9	27	Mixed	30	50	Outpatient (ambulatory); 51 centers, public and private hospitals	Argentina	Some concerns
HFHC (Heart Failure Home Care Trial) (Soran et al. (2008))	[77]	HFHC (Heart Failure Home Care Trial)	STS	-	160	155	6	76.9±7.1	69	HFrEF	24±9	42	Post-discharge	USA	Some concerns
VA TeleHF (Wakefield) (Wakefield 2008)	[73]	VA TeleHF (Wakefield)	STS	3-arm RCT	47	49	12	71.8±10.2	0	Mixed	44	72	Post-discharge	USA	Some concerns
Wakefield 2009 (3-arm) (Wakefield et al. (2009))	[69]	Wakefield 2009 (3-arm)	STS	3-arm RCT	47	49	6	69.0±10.0	1	Mixed	NR	NR	Post-discharge	USA	Some concerns
Tele-HF (Chaudhry et al. (2010))	[82]	Tele-HF	STS	Parallel RCT	826	827	6	61.0	44	Mixed	35	57	Outpatient cardiology practices (33 sites, USA)	USA	Some concerns
SPAN-CHF II (Weintraub et al. (2010))	[67]	SPAN-CHF II	STS	Parallel RCT	95	93	3	69.5±14.2	37	Mixed	32±17	53	Multicenter (4 sites: NEMC, RIH, Lahey, and BIDMC)	USA	Some concerns
CHAT (Krum et al. (2013))	[57]	CHAT	STS	Cluster RCT	139	161	12	73.0±10.0	38	Mixed	37±14	42	Primary care (general practice)	Australia	Some concerns
BEAT-HF (Ong et al. (2016))	[84]	BEAT-HF	STS	Parallel RCT	715	722	6	73.0	47	Mixed	43	75	Hospital (post-discharge)	USA	Low
Negarandeh 2019 (Negarandeh et al. (2019))	[52]	Negarandeh 2019	STS	-	40	40	2	NR	NR	NR	NR	NR	NR	Iran	Some concerns
DAA/Feijó Brazil (Feijó et al. (2021))	[50]	DAA/Feijó Brazil	STS	Parallel RCT	99	107	3	64.0±12.0	42	Mixed	33±12	44	Outpatient (HF clinic, Hospital de Clínicas de Porto Alegre)	Brazil	Some concerns
MESSAGE-HF (Rohde et al. (2024))	[46]	MESSAGE-HF	STS	Parallel RCT	352	347	6	61.3±14.6	32	HFrEF	27	39	Outpatient HF clinics (post-HF hospitalization, enrolled within 30 days of discharge)	Brazil	Some concerns
PRADOC (Roubille et al. (2024))	[45]	PRADOC	STS	-	202	202	12	75.0±12.0	30	Mixed	42±16	90	Post-discharge	France	Some concerns
Ribeiro et al. (2025)	[42]	-	STS	Parallel RCT	70	57	6	63.6±10.7	50	Mixed	NR	NR	Post-discharge	Brazil	Some concerns

**Table 4:** Intervention characteristics by study Based on the TIDieR framework RPM: remote patient monitoring, IHM: implantable hemodynamic monitoring, ICD: implantable cardioverter-defibrillator, RV: right ventricular, PAD: pulmonary artery diastolic pressure, CRT-D: cardiac resynchronization therapy defibrillator, TM: telemonitoring, APN: advanced practice nurse, BP: blood pressure, HR: heart rate, ECG: electrocardiogram, HTM: home telemonitoring, HHA: home health agency, GP: general practitioner, ED: emergency department, SBP: systolic blood pressure, DBP: diastolic blood pressure, TFC: thoracic fluid content, BM: body mass, TBW: total body water, ICG: impedance cardiography, RSM: recommendation support module, MCQ: multiple-choice question, STS: structured telephone support, SOB: shortness of breath, IQR: interquartile range, AHM: automated home monitoring, UCLA: University of California, Los Angeles, CCS: Clinical Congestion Score, PND: paroxysmal nocturnal dyspnea, NYHA: New York Heart Association, SMS: short message service, CHF: congestive heart failure, PA: pulmonary artery, AT/AF: atrial tachycardia/atrial fibrillation, VF: ventricular fibrillation, HR: heart rate, HRV: heart rate variability, VT/AT: ventricular tachycardia/atrial tachycardia, IEGM: intracardiac electrogram, IVR: interactive voice response, LCD: liquid-crystal display, VPN: virtual private network, HTM: home telemonitoring, PHQ-2: Patient Health Questionnaire-2, DM: disease management, UFMG: Universidade Federal de Minas Gerais (Federal University of Minas Gerais, Brazil), TIDieR: Template for Intervention Description and Replication

Study	Reference	RPM category	Subcategory	Technology/platform	Monitoring parameters	Frequency	Who responds	Alert threshold	Patient education	Duration (week)	Adherence (%)
CHAMPION (Abraham et al. (2011))	[6]	Invasive	Implanted PA sensor	CardioMEMS PA pressure sensor	PA pressure (systolic, diastolic, and mean)	Daily	Cardiologist	Yes	No	26	NR
REDUCEhf (Adamson et al. (2011))	[34]	Invasive	ICD remote (IHM)	Medtronic Chronicle IHM-ICD (implantable hemodynamic monitor + ICD)	RV systolic/diastolic pressure, estimated PAD, dP/dt, heart rate, and activity (continuous)	Automatic (weekly upload via telephone)	Cardiologist	Yes	Yes	52	NR
EVOLVO (Landolina et al. (2012))	[33]	Invasive	ICD remote	Medtronic CareLink Network (wireless ICD/CRT-D; full interrogation via telephone; secure web platform; OptiVol intrathoracic impedance)	Intrathoracic impedance (OptiVol), AT/AF burden, ICD shocks, ventricular rate, lead impedances, VF detection status, battery, daily activity, HR, HRV	Automatic	Cardiologist	Yes	Yes	70	NR
IN-TIME (Hindricks et al. (2014))	[30]	Invasive	ICD/CRT-D remote monitoring	Remote monitoring (home monitoring and ICD/CRT-D)	Arrhythmias (VT/AT), biventricular pacing %, ventricular extrasystole frequency, patient activity trend, intracardiac electrogram, pacing/impedance safety notifications	Daily automatic transmission (typically 03:00h) + on tachyarrhythmia detection	Investigational site physician + central monitoring unit (nurses + physicians at Heart Center Leipzig); patient contact by phone within 48 hours of alert	Pre-defined: VT/shock, AT onset/burden, CRT <80% over 48 hours, VES >110/hour or rising, activity decline, abnormal IEGM, pacing/impedance alerts, transmission gap >3 days	Standardized telephone interview on contact: symptoms, drug adherence, weight gain >2 kg/3 days	52	85%
CONNECT-OptiVol (Lüthje et al. (2015))	[38]	Invasive	ICD remote	Remote ICD/CRT-D monitoring with OptiVol fluid index	Intrathoracic impedance (OptiVol fluid index); Cardiac Compass; remote transmission via CareLink network	Continuous automatic transmission on alert; otherwise, routine remote check	Study investigator team (physician review of transmitted report)	OptiVol threshold set to nominal 60 Ω at implantation (unchanged during follow-up); audible alert disabled in both groups	Patient phones investigator on audible alert disabled; remote arm: weight monitoring daily after diuretic increase	65	NR
OptiLink HF (Böhm et al. (2016))	[37]	Invasive	ICD remote	Medtronic OptiVol intrathoracic impedance fluid index + CareLink wireless network (CareAlert automatic text to physician)	Intrathoracic fluid index (OptiVol impedance); ICD device diagnostics	Automatic	Cardiologist	Yes	No	97	76%
TELECART (Sardu et al. (2016))	[36]	Invasive	CRT-D remote	Biotronik Home Monitoring (Lumax 640HF/Iforia CRT-D): daily automatic IEGM + arrhythmia telemetry	Device diagnostics: biventricular pacing %, arrhythmia episodes (AF/VT/VF), ICD shocks, intracardiac electrograms, patient activity	Continuous automatic transmission; alerts reviewed on working days; site must confirm within 48 hours	Central monitoring unit (trained nurses + physicians) at Giovanni Paolo II Foundation; alerts forwarded to the investigational site; investigators contact patient by telephone	Predefined medical events: VT/AF episodes, low biventricular pacing %, increased ventricular extrasystoles, decreased patient activity, abnormal IEGM	Patients instructed to monitor weight, dyspnea, and symptoms; telephone interview at alert: weight gain >2 kg/3 days, dyspnea, and drug adherence	52	NR
MORE-CARE (Boriani et al. (2017))	[31]	Invasive	CRT-D remote	Medtronic CareLink remote monitor (wireless home unit; Medtronic CRT-D with wireless transmission)	Lung fluid accumulation (OptiVol), atrial tachyarrhythmia (AF/flutter), system integrity, ECG/device diagnostics	Automatic (remote checks every 4 months alternating with in-office; automatic alerts continuous)	Cardiologist	Yes	Yes	96	83%
RESULT (Tajstra et al. (2020))	[32]	Invasive	ICD remote/CRT-D remote	Multi-manufacturer (Carelink/Merlin/LATITUDE/home monitoring); centralized remote monitoring office	Device diagnostics (arrhythmia, system integrity, battery, charge time, and VF therapy); telephone interview (dyspnea, drug compliance, and weight)	Automatic daily transmission	Centralized team (2 physicians + 2 EP nurses)	Yes	Yes	52	86%
GUIDE-HF (Lindenfeld et al. (2021))	[8]	Invasive	Implanted PA sensor	CardioMEMS PA pressure sensor	PA systolic pressure, PA diastolic pressure, PA mean pressure, and heart rate	Daily uploads	Clinician (investigator)	PA pressure elevation triggers diuretic/vasodilator titration per protocol	Yes: scripted calls every 2 weeks (first 3 months), then monthly; masked caller	52	85%
MONITOR-HF (Brugts et al. (2023))	[7]	Invasive	Invasive hemodynamic (pulmonary artery pressure)	CardioMEMS PA pressure sensor (Abbott)	Pulmonary artery pressure (daily uploads)	Daily patient uploads (84.3% adherence)	HF nurse/clinician team; dedicated outpatient clinic nurses	PA pressure thresholds: diuretics titrated for hypervolemia/hypovolemia; vasodilators for increased vascular resistance	Yes: patient training on PA pressure uploads	192	84%
Jerant 2001 (3-arm) (Jerant et al. (2001))	[81]	Non-invasive TM	mHealth app	2-way video-conference device with integrated electronic stethoscope	Video clinical assessment, electronic auscultation (heart/lung sounds), and symptom review	Variable	Nurse	NR	Yes	26	NR
Benatar et al. (2003)	[87]	Non-invasive TM	Weight + BP + HR + SpO2 (daily Internet transmission)	Transtelephonic home monitoring devices	Weight, blood pressure, heart rate, and oxygen saturation (SpO2)	Daily transmission to secure Internet site	APN collaborating with cardiologist	Clinical guideline-based thresholds for medication titration	Yes: APN telephoned patients and provided education and medication adjustments per HF clinical guidelines	13	NR
Jerant 2003 (3-arm) (Jerant et al. (2003))	[79]	Non-invasive TM	mHealth app	Video home telecare (2-way videoconference unit) + structured telephone monitoring (telephone arm)	CHF symptoms, weight, self-care adherence, medications, and health status; video clinical assessment + electronic stethoscope	Variable	Nurse	NR	Yes	26	NR
TEN-HMS (Cleland et al. (2005))	[85]	Non-invasive TM	Weight + BP + HR + ECG rhythm (twice-daily automated transmission)	HTM: daily BP/weight/ECG; nurse-led telephonic review	Weight, blood pressure, heart rate, and single-lead ECG rhythm	Twice daily (before breakfast and before evening meal)	Specialist nurse (reviews alerts and contacts patient/primary care physician)	Weight change >2 kg; HR <50 or >80 bpm; new arrhythmia; SBP <90 or >140 mmHg	Individualized written management plan; device installation instruction; nurse available by telephone	34	81%
Antonicelli et al. (2008)	[76]	Non-invasive TM	Nurse telephone	A&D UA-767 PC (BP device) + Card Guard CG-7100 (12-lead transtelephonic ECG, Card Guard Scientific Survival Ltd., Israel)	BP, HR, body weight, 24-hour urine output, ECG (weekly 12-lead transtelephonic), symptoms (dyspnea and edema), and medication adherence	Weekly	Mixed	Yes	Yes	52	91%
Dansky 2008 (Dansky and Vasey 2008)	[74]	Non-invasive TM	Non-invasive TM	HomMed Health Monitor/ViTel Net (1-way asynchronous) + Aviva (2-way video + stethoscope)	Weight, BP, HR, SpO2, and blood glucose (as ordered); symptom assessment (Omaha PRSO: diet/fluid intake, physical activity, and medication effectiveness)	Daily (one-way); 2-3×/week (video arm)	Nurse (HHA central station)	Yes	No	9	NR
Kashem 2008 (Kashem et al. (2008))	[75]	Non-invasive TM	mHealth app	InSight Telehealth system	Weight, BP, HR, SpO2, and symptoms	Daily	Nurse	Yes	Yes	52	NR
Dansky 2009 (Health Buddy) (Dansky et al. (2009))	[68]	Non-invasive TM	IVR	Health Buddy interactive device (patient-entered data to care manager)	Daily weight, ankle swelling, and SOB (5-item symptom questionnaire)	Daily	Care manager	Yes	Yes	26	NR
Home-HF (Dar et al. (2009))	[71]	Non-invasive TM	Home device	Honeywell HomMed	Weight, BP, HR, SpO2, and 4-symptom questions (breathlessness, orthopnea, dizziness, and ankle swelling)	Daily (Monday-Friday nurse review)	HF nurse	Yes	Yes	26	95%
Giordano et al. (2009)	[88]	Non-invasive TM	Portable ECG device + nurse teleconsultation	Portable device transmitting 1-lead ECG trace by telephone	1-lead ECG trace	NR (abstract does not specify frequency)	Nurse (interactive teleconsultation at receiving station)	NR (abstract does not specify thresholds)	NR	52	NR
HHH (Mortara et al. (2009))	[70]	Non-invasive TM	Home device (IVR + cardiorespiratory recorder)	NICRAM system (IVR vital signs + portable Holter cardiorespiratory recorder + digital BP + scale)	Weight, HR, systolic BP, dyspnea score, asthenia score, edema score, therapy changes, and blood results (weekly); 24-hour cardiorespiratory recording monthly (strategies 2+3)	Weekly (vital signs); monthly (telephone + cardiorespiratory)	Monitoring nurse or physician (after automated alert)	Yes	Yes	48	81%
MOBITEL (Scherr et al. (2009))	[72]	Non-invasive TM	mHealth app	Nokia 3510 mobile phone + Soehnle Creta Scale + BosoMedicus BP device + MOBITEL web platform (Zope/Interbase; AIT Graz)	Blood pressure, heart rate, body weight, and HF medication dose	Daily	Cardiologist	Yes	Yes	26	95%
Tompkins 2010 (Tompkins and Orwat 2010)	[65]	Non-invasive TM	Wearable	Vital signs TM (daily to central nursing station)	Weight, blood pressure, heart rate, SpO2, and weekly symptom prompts	Daily	Central nursing station	Yes	Yes	26	NR
TIM-HF (Koehler et al. (2011))	[63]	Non-invasive TM	Wearable	ECG + BP + weight daily; physician-led telemedical center	ECG (3-lead), blood pressure, and body weight	Daily	Physician (24/7 telemedical center)	Predefined standard operating procedures; physician contacts patient when threshold triggered or patient-initiated	Device training provided within 5 working days of randomization	~113	81%
THCM (Wade 2011) (Wade et al. (2011))	[62]	Non-invasive TM	Wearable	Internet-connected telemonitoring with nurse case management alerts	Weight, BP, medication adherence, and symptom questions	Daily (weekdays)	Nurse	Yes	Co-intervention	26	74%
TEHAF (Boyne et al. (2012))	[61]	Non-invasive TM	IVR	Health Buddy device (LCD + 4 keys, landline phone); daily pre-set tailored dialogues on symptoms/knowledge/behavior; no automatic vital sign transfer	Symptoms (HF-related), knowledge, and behavior/adherence; BP and HR collected at face-to-face visits only	Daily (dialogues sent daily)	Heart failure nurse + nurse assistant (reviewed responses on desktop; immediate response to positive symptom answers)	Risk profiles (low/medium/high) generated from responses; positive symptom answers triggered immediate nurse response	Yes: tailored education component (4 programs varying symptom monitoring + education intensity)	52	90%
TEMA-HF 1 (Dendale et al. (2012))	[86]	Non-invasive TM	Wearable	Electronic body weight scale + blood pressure monitor + cell phone (Bluetooth to central computer)	Weight, blood pressure, and heart rate	Daily	GP + HF nurse/specialist (collaborative)	Yes (weight: ±2 kg, SBP: 90-140, HR: 50-90 bpm)	Yes	26	83%
Gámez-López 2012 (Gámez-López et al. (2012))	[59]	Non-invasive TM	Nurse telephone	Structured telephone support by nurse (monthly calls + symptom-triggered calls)	Symptoms, weight, and functional status	Monthly + as needed	Nurse	Yes	Yes	47	NR
Seto 2012 (Seto et al. (2012))	[60]	Non-invasive TM	mHealth app	Mobile phone with automated wireless transmission + alerts to cardiologist	Daily weight, daily BP, weekly single-lead ECG, and daily symptom questionnaire	Daily (weight, BP, and symptoms); weekly (single-lead ECG)	Cardiologist (receives email/mobile alerts, contacts patients by phone, and adjusts medications and schedules clinic visits)	Clinician-defined per-patient physiological thresholds; low-priority (retake) to high-priority (go to ED/call 911) alerts sent to cardiologist's mobile phone	Yes: individual training session on system use; automated instructions sent to patient's mobile phone; adherence reminder calls at 10 am	26	84%
Delaney 2013 (Delaney et al. (2013))	[58]	Non-invasive TM	Automated vital signs monitor	HomMed Health Monitor (Honeywell): automated vital signs via phone lines; daily transmission to central server	Weight, BP, HR, SpO2, and symptoms (5 daily questions)	Daily	TM program manager (daily review)	Yes	Co-intervention	13	NR
Pedone 2015 (Pedone et al. (2015))	[55]	Non-invasive TM	Non-invasive multiparameter home device	A&D Engineering sphygmomanometer + scale + Nonin Medical pulse oximeter; Android smartphone transmitter; web-based monitoring portal + geriatrician telephone support	SpO2, heart rate, blood pressure, and body weight	Daily (weight 1×/day; BP + HR 2×/day; SpO2 3×/day)	Geriatrician	Yes	Yes	26	62%
TIM-HF2 (Koehler et al. (2018))	[5]	Non-invasive TM	RPM with physician-led telemedical center	Daily ECG + blood pressure + weight via mobile device	Daily ECG (3-channel, 2 minutes or streaming; PhysioMem PM1000 GETEMED), blood pressure (UA767PBT A&D), body weight (Seca 861), SpO2 (Masimo SET), and self-rated health status (1-5 scale)	Daily transmission via mobile phone network (VPN tunnel) to telemedical center at fixed time	Telemedical center physicians + HF nurses 24/7, Monday-Sunday; Fontane software (CE-marked) with risk algorithms; patient GP and cardiologist also involved	Risk categorization (low/high) using MR-proADM + transmitted vital signs; reassessed every 3 months; Fontane algorithms triggered alerts	Yes: patient education program initiated at device setup by certified nurses; monthly structured telephone interviews throughout study	52	97%
Renewing Health (Olivari 2018) (Olivari et al. (2018))	[54]	Non-invasive TM	mHealth app	RENEWING HEALTH platform (European CIP ICT PSP project, telemedicine home monitoring system)	NR (standard HF parameters; RENEWING HEALTH platform)	Daily	Mixed	Yes	Co-intervention	52	83%
Pekmezaris 2019 (Pekmezaris et al. (2019))	[53]	Non-invasive TM	mHealth app	American TeleCare LifeView (home-installed video telehealth + daily vital signs)	Weight, BP, SpO2, and HR/pulse	Daily	Nurse	Yes	Co-intervention	13	50%
OSICAT (Galinier et al. (2020))	[51]	Non-invasive TM	Wearable	Electronic scale (body weight daily) + 8-symptom questionnaire device (daily); automated expert system generates alerts; secure server	Daily body weight, daily HF symptom questionnaire (8 items), and personalized education (phone calls every 3 weeks)	Daily	Nurse (working days only); nurse contacts patient to validate alert; if appropriate, advises patient to contact GP/cardiologist; follow-up call 48 hours later	Automated expert system thresholds (not specified in paper); alerts generated based on weight/symptom data	Yes: personalized info pack + telephone calls every 3 weeks with HF-specialized nurse; topics: disease knowledge, medications, sign recognition, and lifestyle (diet + activity)	78	60%
Yanicelli 2021 (Yanicelli et al. (2021))	[49]	Non-invasive TM	mHealth app	Custom HTM app (daily measurements + alerts)	Weight, blood pressure, heart rate, and symptoms (ankle/leg swelling and dyspnea)	Daily	Nurse	Yes	Yes	13	NR
Johnson 2022 (Johnson et al. (2022))	[48]	Non-invasive TM	mHealth app	mHealth HF self-care app (knowledge, self-efficacy, and symptom detection)	Symptom detection, self-management education, knowledge, and self-efficacy; no physiological sensor data transmitted	Daily app use (post-discharge)	Clinical escalation process available; no escalations required during study	Clinical escalation process built in (no triggers fired)	Yes: core component: HF knowledge, self-care skills, and symptom recognition	12	88%
AMULET (Krzesiński et al. (2022))	[47]	Non-invasive TM	Nurse telephone	Web telemedicine + nurse-led non-invasive assessments + remote cardiologist decisions	HR, SBP, DBP, TFC, BM, and TBW, via ICG (Cardioscreen 2000) + bioimpedance (Tanita MC-418MA)	7 scheduled outpatient visits over 12 months	Remote cardiologist (via RSM)	Color-coded RSM alarms: white (optimal), green/yellow/red (staged); red TFC = urgent in-person consult within 2 hours	Symptom monitoring questionnaire; recommendation provided at each visit	52	86%
ControlVit (Achury-Saldaña et al. (2024))	[44]	Non-invasive TM	mHealth app	ControlVit mobile app (daily weight/BP/HR + 8-item symptom questionnaire)	Weight, BP, and HR + 8-item symptom questionnaire	Daily	Nurse (daily web platform review + real-time alerts)	Yes	Co-intervention	26	NR
Arnar 2025 (Arnar et al. (2025))	[43]	Non-invasive TM	mHealth app	Sidekick Health digital therapeutics smartphone app (RPM + self-care + education + lifestyle support)	Symptoms (breathlessness, fatigue, leg edema, chest pain, and dizziness: 5 daily MCQ); weight; vital signs (optional, own devices); step count (automatic via smartphone)	Daily (weeks 1-12), tapering to weekly (weeks 24-52); daily optional in maintenance	Nurses: reviewed RPM data 3×/day during working hours (weekdays only); traffic-light algorithm (green/yellow/red) guided response	Three-tiered traffic light: green=stable, yellow=increased monitoring, red=immediate nurse contact	Yes: 24-week educational program (nutrition, medication adherence, sleep, exercise, stress, mental health, and HF knowledge); video + content cards; recap weeks 25-48	48	93%
M-Cardio (ERICA-HF) (Rustambekova et al. (2025))	[41]	Non-invasive TM	mHealth app	M-Cardio app (Android; custom-developed)	Dyspnea, body position, palpitations, edema, body weight, BP, and HR (7 items)	Twice weekly (daily if necessary)	Supervising physician (via WhatsApp/phone when >2 values deviate)	Yes	Yes	48	NR
Vietnam HFrEF RCT (Tran et al. (2025))	[40]	Non-invasive TM	Nurse telephone + mHealth app	Telephone follow-ups + HTM (BP/HR/weight); STS + non-invasive TM hybrid	Weight, BP, HR, and symptoms (dyspnea, cough, edema, and palpitations)	Daily	Mixed	Yes	Co-intervention	26	NR
TeleMedBot (Zheleznykh et al. (2025))	[39]	Non-invasive TM	mHealth app	TeleMedBot (custom; Python REST API + PostgreSQL; patient-facing via smartphone messenger)	BP, HR, dyspnea, body position, palpitations, edema, and body weight	Daily	Cardiologist	Yes	Yes	26	76%
Riegel et al. (2002)	[80]	STS	Nurse telephone	Pfizer "At Home With Heart Failure," a computer-supported telephonic case management software	Symptoms, weight, fluid retention, SOB, medication adherence, diet adherence, and functional status	Variable	Nurse	Yes	Yes	26	NR
Laramee et al. (2003)	[78]	STS	Nurse telephone	4-component CHF case management: discharge planning + comprehensive patient education + 12-week telephone follow-up + optimal medication promotion; educational materials: "Heartworks" booklet, weight logs, sodium guide, and home scales	CHF symptoms, daily weight, edema, medications, self-care, fluid/sodium, laboratory values, and appointments	Variable	Nurse	Yes	Yes	12	NR
DIAL (GESICA investigators 2005)	[83]	STS	Nurse telephone monitoring + education + counselling	Nurse telephone follow-up (structured)	Symptoms, medication adherence, weight, dietary compliance, and functional class	Fortnightly first 4 calls, then individualized by nurse algorithm	Trained nurse (can adjust diuretic dose and recommend unscheduled visits)	Nurse discretion based on structured call data; algorithm-driven intervals	Yes: education booklet at randomization; ongoing counselling at each call	96	NR
HFHC (Heart Failure Home Care Trial) (Soran et al. (2008))	[77]	STS	IVR (automated telephone)	Alere DayLink HF Monitoring System (electronic scale + IVR symptom questionnaire + nurse review)	Weight, SOB at night, extra pillow use, ankle edema, fatigue, and cough (symptom IVR daily)	Daily (nurse review 7 days/week, 365 days/year)	Nurse (alerts faxed to primary care physician)	Yes	Yes	24	97%
VA TeleHF (Wakefield) (Wakefield et al. (2008))	[73]	STS	Nurse telephone	Personal home telephone (primary arm); CyberCare EHC 200 Sentinel/TeleVyou 500SP videophone (secondary arm)	Symptoms (checklist), daily weight, blood pressure, and ankle circumference	Daily contact 3× first week post-discharge, then weekly 11× weeks (14 contacts total over 3 months)	Registered nurse (study nurse)	Yes	Yes: discharge plan review, symptom checklist, and behavioral compliance strategies	12	80%
Wakefield 2009 (3-arm) (Wakefield et al. (2009))	[69]	STS	Nurse telephone	Telephone calls (home); weekly nurse-initiated calls; maximum of 14 contacts over 90 days; videophone arm (secondary): EHC 200 Sentinel/TeleVyou 500SP videophone	Symptom review checklist (weight gain and decompensation symptoms), medication compliance, self-efficacy, and patient education	Weekly	Nurse	Yes	Yes	13	94%
Tele-HF (Chaudhry et al. (2010))	[82]	STS	IVR	Automated telephone interactive voice-response system (daily symptoms + weight)	Daily symptoms (dyspnea, weight gain, fatigue, edema, and general health) + weight via telephone keypad entry; PHQ-2 depression screen every 30 days	Daily calls (patients call toll-free number)	Site coordinators review daily on secure Internet portal (weekdays only, excluding holidays); cardiologists make clinical decisions on variances	Predetermined variance thresholds for each question; variances flagged for clinician attention; 29,163 total variances (median: 21/patient, IQR: 5-54)	Educational materials from Heart Failure Society of America; training on IVR system; scale and telephone provided if needed; reminder calls for non-adherence	26	55%
SPAN-CHF II (Weintraub et al. (2010))	[67]	STS	AHM (weight/BP/HR) + symptom questionnaire	AHM: weight/BP/HR, added to telephonic DM	Body weight, blood pressure, heart rate, and patient self-assessment	Daily (AHM transmission)	HF nurse manager (Monday-Friday, daily review; 24/7 on-call)	Prespecified thresholds for weight, BP, pulse, and symptoms; nurse calls patient if exceeded	Yes: enrollment visit education on HF self-monitoring, diet, and medication; Health Buddy daily questionnaire	13	NR
CHAT (Krum et al. (2013))	[57]	STS	IVR	Trained cardiac nurse telephone follow-up (TeleWatch software)	Symptoms, weight, fluid intake, medication adherence, stress, depression, exercise, smoking, and alcohol (via TeleWatch IVR)	Monthly minimum (automated calls); unscheduled calls patient-initiated at any time	Trained cardiac nurse	Prespecified symptom/sign alerts via TeleWatch Patient Watch Screen → nurse follow-up	Yes: action plan for detecting deterioration; TeleWatch IVR includes education modules on HF self-management	52	66%
BEAT-HF (Ong et al. (2016))	[84]	STS + non-invasive TM	Nurse telephone + wearable	Health coaching telephone calls + telemonitoring (daily BP/HR/symptoms/weight)	Weight, blood pressure, heart rate, and symptoms (3 daily questions; Bluetooth devices)	Daily TM; 9 coaching calls over 6 months (weekly during the first month, then monthly)	Registered nurses at telephone call center (UCLA-based)	Yes: predetermined thresholds for weight/BP/HR; triggers nurse telephone callback	Yes: pre-discharge HF education (booklet; teach-back method; low health literacy design)	26	55%
Negarandeh 2019 (Negarandeh et al. (2019))	[52]	STS	-	Telephone-based monitoring	-	-	-	-	-	NR	NR
DAA/Feijó Brazil (Feijó et al. (2021))	[50]	STS	Nurse telephone	Nurse telephone calls: diuretic algorithm protocol	Body weight (daily, morning pre-meal), CCS (0-22), dyspnea, PND, orthopnea, peripheral edema, NYHA functional class, and furosemide dose adjustment	Weekly (1-2×/week for 30 days)	Nurse (algorithm-guided diuretic titration)	Yes	Yes: co-intervention	4	NR
MESSAGE-HF (Rohde et al. (2024))	[46]	STS	mHealth (SMS-based bidirectional telemonitoring)	Bidirectional SMS automated text messaging; 4 daily SMS; red flags → diuretic adjustment or HF team telephone call	Self-care behaviors, symptoms, and adherence; red-flag → diuretic adjustment or callback	4 SMS/day	Nurse/team (callback on red-flag alerts)	Weight gain ≥2 kg/1st week or ≥3 kg/1st month; nocturnal dyspnea 2 consecutive nights; non-adherence to medications 2 consecutive days	Yes: 4 SMS/day included educational messages on HF signs/symptoms, daily activities, lifestyle, medication, and fluid intake	4	75%
PRADOC (Roubille et al. (2024))	[45]	STS	Nurse telephone	Administrative transition care coordination (PRADO-IC national program)	Post-discharge cardiology + GP follow-up scheduling	Weekly	Nurse	No	Co-intervention	24	NR
Ribeiro et al. (2025)	[42]	STS	Nurse telephone	UFMG-developed custom software; standard SMS; BP cuff + scale provided; decision trees with 5 pathways (emergency, same-day teleconsult, elective teleconsult ≤7 days, diuretic adjustment, and maintenance)	Weight (daily), blood pressure (daily), heart rate (daily), decompensation symptoms, medication adherence, and NYHA class	Weekly	Mixed	Yes	Co-intervention	26	66%

Risk of Bias

**Figure 2:**
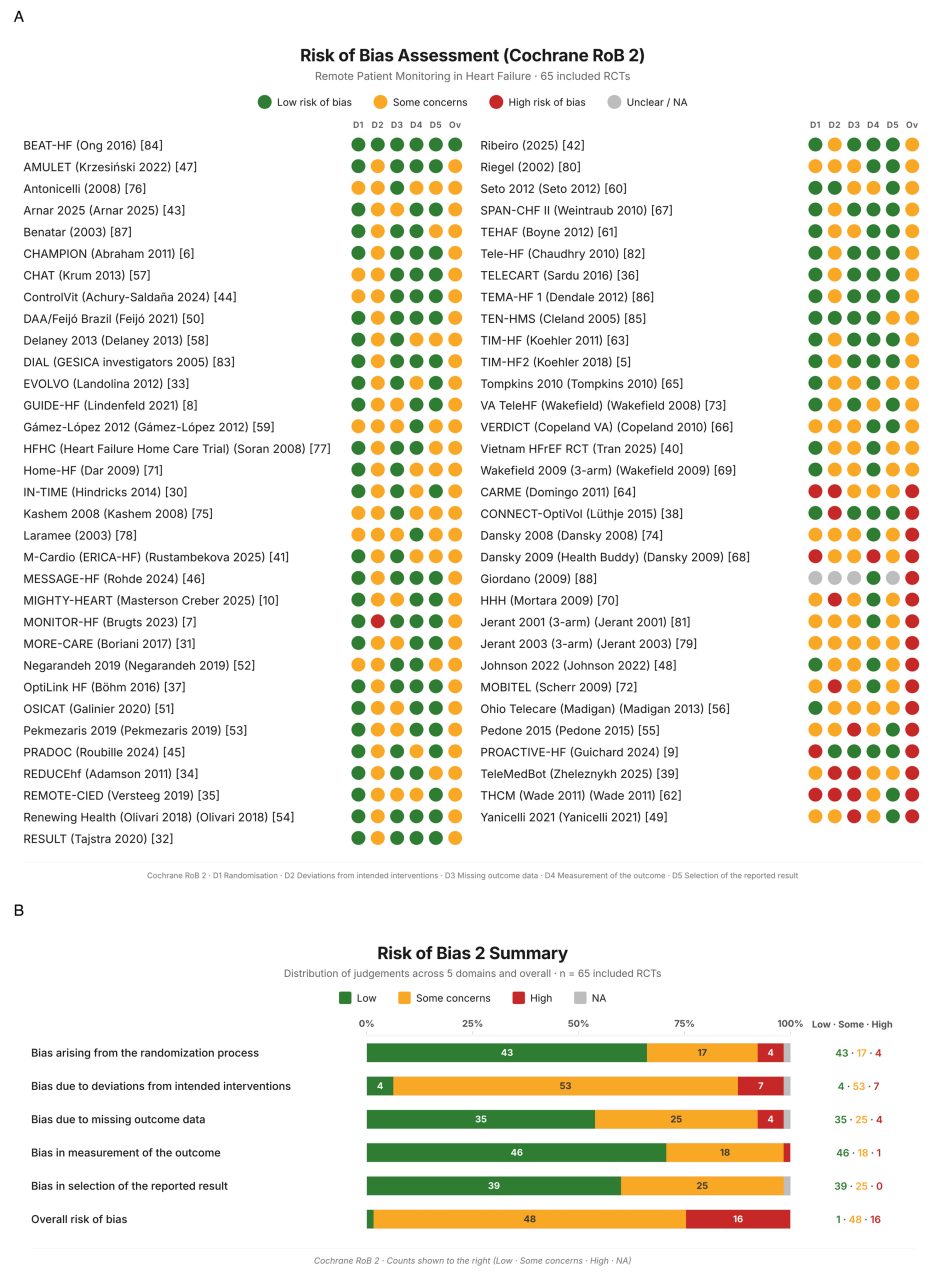
Risk of bias 2 assessment (A) Per-study traffic-light judgements across five domains plus overall, sorted by overall judgement (low → high; n=65 RCTs). (B) Domain-level distribution of judgements as percentages. RCT: randomized controlled trial

The majority of studies (48 of 65) were judged as having "some concerns" for overall risk of bias, primarily driven by the open-label design inherent to RPM interventions (D2: deviations from intended interventions). Sixteen studies were rated as high risk of bias, and one as low risk. Per-study judgements and domain-level distributions are shown in Figure 2, and detailed RoB 2 assessments with notes for every study are reported in Table 5.

**Table 5:** Per-study risk of bias 2 judgements with notes (Reference column gives reference numbers)

Study	Reference	D1: Randomization	D2: Deviations	D3: Missing data	D4: Measurement	D5: Selection	Overall
BEAT-HF (Ong et al. (2016))	[84]	Low	Low	Low	Low	Low	Low
AMULET (Krzesiński et al. (2022))	[47]	Low	Some concerns	Low	Low	Low	Some concerns
Antonicelli et al. (2008)	[76]	Some concerns	Some concerns	Low	Some concerns	Some concerns	Some concerns
Arnar et al. (2025)	[43]	Low	Some concerns	Some concerns	Low	Low	Some concerns
Benatar et al. (2003)	[87]	Low	Some concerns	Low	Low	Some concerns	Some concerns
CHAMPION (Abraham et al. (2011))	[6]	Low	Some concerns	Low	Low	Low	Some concerns
CHAT (Krum et al. (2013))	[57]	Some concerns	Some concerns	Low	Low	Low	Some concerns
ControlVit (Achury-Saldaña et al. (2024))	[44]	Some concerns	Some concerns	Low	Low	Low	Some concerns
DAA/Feijó Brazil (Feijó et al. (2021))	[50]	Low	Some concerns	Low	Low	Low	Some concerns
DIAL (GESICA investigators 2005)	[83]	Low	Some concerns	Low	Low	Low	Some concerns
Delaney 2013 (Delaney et al. (2013))	[58]	Low	Some concerns	Low	Some concerns	Some concerns	Some concerns
EVOLVO (Landolina et al. (2012))	[33]	Low	Some concerns	Low	Some concerns	Low	Some concerns
GUIDE-HF (Lindenfeld et al. (2021))	[8]	Low	Some concerns	Some concerns	Low	Low	Some concerns
Gámez-López 2012 (Gámez-López et al. (2012))	[59]	Some concerns	Some concerns	Some concerns	Low	Some concerns	Some concerns
HFHC (Heart Failure Home Care Trial) (Soran et al. (2008))	[77]	Low	Some concerns	Low	Low	Some concerns	Some concerns
Home-HF (Dar et al. (2009))	[71]	Low	Some concerns	Low	Some concerns	Some concerns	Some concerns
IN-TIME (Hindricks et al. (2014))	[30]	Low	Some concerns	Low	Some concerns	Low	Some concerns
Kashem 2008 (Kashem et al. (2008))	[75]	Some concerns	Some concerns	Low	Some concerns	Some concerns	Some concerns
Laramee et al. (2003)	[78]	Some concerns	Some concerns	Some concerns	Low	Some concerns	Some concerns
M-Cardio (ERICA-HF) (Rustambekova et al. (2025))	[41]	Low	Some concerns	Low	Some concerns	Some concerns	Some concerns
MESSAGE-HF (Rohde et al. (2024))	[46]	Low	Some concerns	Low	Low	Low	Some concerns
MIGHTY-HEART (Masterson Creber et al. (2025))	[10]	Low	Some concerns	Some concerns	Low	Low	Some concerns
MONITOR-HF (Brugts et al. (2023))	[7]	Low	High	Low	Low	Low	Some concerns
MORE-CARE (Boriani et al. (2017))	[31]	Low	Some concerns	Some concerns	Low	Low	Some concerns
Negarandeh 2019 (Negarandeh et al. (2019))	[52]	Some concerns	Some concerns	Low	Low	Some concerns	Some concerns
OSICAT (Galinier et al. (2020))	[51]	Low	Some concerns	Some concerns	Low	Low	Some concerns
OptiLink HF (Böhm et al. (2016))	[37]	Low	Some concerns	Low	Low	Low	Some concerns
PRADOC (Roubille et al. (2024))	[45]	Low	Some concerns	Low	Some concerns	Low	Some concerns
Pekmezaris 2019 (Pekmezaris et al. (2019))	[53]	Low	Some concerns	Some concerns	Low	Low	Some concerns
REDUCEhf (Adamson et al. (2011))	[34]	Low	Some concerns	Low	Low	Some concerns	Some concerns
REMOTE-CIED (Versteeg et al. (2019))	[35]	Low	Some concerns	Some concerns	Some concerns	Low	Some concerns
RESULT (Tajstra et al. (2020))	[32]	Low	Some concerns	Low	Low	Low	Some concerns
Renewing Health (Olivari 2018) (Olivari et al. (2018))	[54]	Low	Some concerns	Low	Low	Low	Some concerns
Ribeiro et al. (2025)	[42]	Low	Some concerns	Low	Low	Low	Some concerns
Riegel et al. (2002)	[80]	Some concerns	Some concerns	Some concerns	Low	Low	Some concerns
SPAN-CHF II (Weintraub et al. (2010))	[67]	Low	Some concerns	Low	Low	Low	Some concerns
Seto 2012 (Seto et al. (2012))	[60]	Low	Low	Some concerns	Some concerns	Low	Some concerns
TEHAF (Boyne et al. (2012))	[61]	Low	Some concerns	Some concerns	Low	Low	Some concerns
TELECART (Sardu et al. (2016))	[36]	Low	Some concerns	Low	Low	Low	Some concerns
TEMA-HF 1 (Dendale et al. (2012))	[86]	Low	Some concerns	Low	Low	Low	Some concerns
TEN-HMS (Cleland et al. (2005))	[85]	Low	Low	Low	Low	Some concerns	Some concerns
TIM-HF (Koehler et al. (2011))	[63]	Low	Some concerns	Low	Low	Low	Some concerns
TIM-HF2 (Koehler et al. (2018))	[5]	Low	Some concerns	Low	Low	Low	Some concerns
Tele-HF (Chaudhry et al. (2010))	[82]	Low	Some concerns	Low	Low	Low	Some concerns
Tompkins 2010 (Tompkins and Orwat (2010))	[65]	Low	Some concerns	Some concerns	Low	Some concerns	Some concerns
VA TeleHF (Wakefield) (Wakefield et al. (2008))	[73]	Low	Some concerns	Low	Some concerns	Low	Some concerns
VERDICT (Copeland VA) (Copeland et al. (2010))	[66]	Some concerns	Some concerns	Some concerns	Low	Low	Some concerns
Vietnam HFrEF RCT (Tran et al. (2025))	[40]	Low	Some concerns	Some concerns	Low	Some concerns	Some concerns
Wakefield 2009 (3-arm) (Wakefield et al. (2009))	[69]	Low	Some concerns	Some concerns	Low	Some concerns	Some concerns
CARME (Domingo et al. (2011))	[64]	High	High	Some concerns	Some concerns	Some concerns	High
CONNECT-OptiVol (Lüthje et al. (2015))	[38]	Low	High	Low	Low	Low	High
Dansky 2008 (Dansky and Vasey (2008))	[74]	Some concerns	Some concerns	Some concerns	Low	Some concerns	High
Dansky 2009 (Health Buddy) (Dansky et al. (2009))	[68]	High	Some concerns	Some concerns	High	Some concerns	High
Giordano et al. (2009)	[88]	Unclear	Unclear	Unclear	Low	Unclear	High
HHH (Mortara et al. (2009))	[70]	Some concerns	High	Some concerns	Low	Some concerns	High
Jerant 2001 (3-arm) (Jerant et al. (2001))	[81]	Some concerns	Some concerns	Some concerns	Low	Some concerns	High
Jerant 2003 (3-arm) (Jerant et al. (2003))	[79]	Some concerns	Some concerns	Some concerns	Some concerns	Some concerns	High
Johnson 2022 (Johnson et al. (2022))	[48]	Low	Some concerns	Some concerns	Low	Some concerns	High
MOBITEL (Scherr et al. (2009))	[72]	Some concerns	High	Some concerns	Low	Some concerns	High
Ohio Telecare (Madigan) (Madigan et al. (2013))	[56]	Low	Some concerns	Some concerns	Some concerns	Some concerns	High
PROACTIVE-HF (Guichard et al. (2024))	[9]	High	Low	Low	Low	Low	High
Pedone 2015 (Pedone et al. (2015))	[55]	Some concerns	Some concerns	High	Some concerns	Low	High
THCM (Wade 2011) (Wade et al. (2011))	[62]	High	High	High	Some concerns	Low	High
TeleMedBot (Zheleznykh et al. (2025))	[39]	Some concerns	High	High	Some concerns	Some concerns	High
Yanicelli 2021 (Yanicelli et al. (2021))	[49]	Some concerns	Some concerns	High	Some concerns	Low	High

Primary Outcomes: All-Cause Mortality

**Figure 3:**
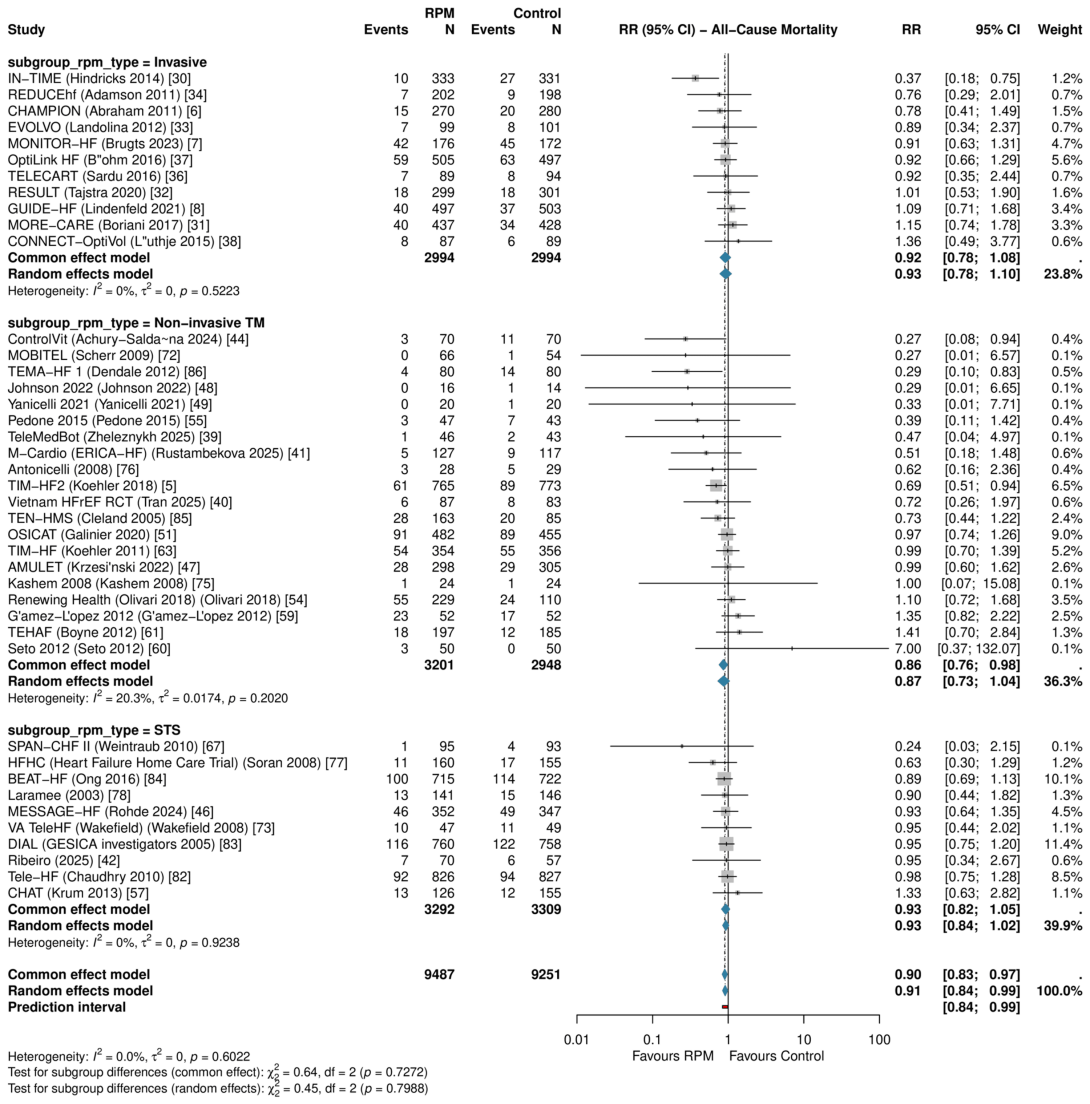
Forest plot of all-cause mortality RR with 95% CI from random-effects meta-analysis (REML + HKSJ). The diamond represents the pooled estimate (RR: 0.911, 95% CI: 0.842-0.985; k=41). RR: risk ratio, CI: confidence interval, REML: restricted maximum likelihood, HKSJ: Hartung-Knapp-Sidik-Jonkman, RPM: remote patient monitoring, TM: telemonitoring, STS: structured telephone support

**Figure 4:**
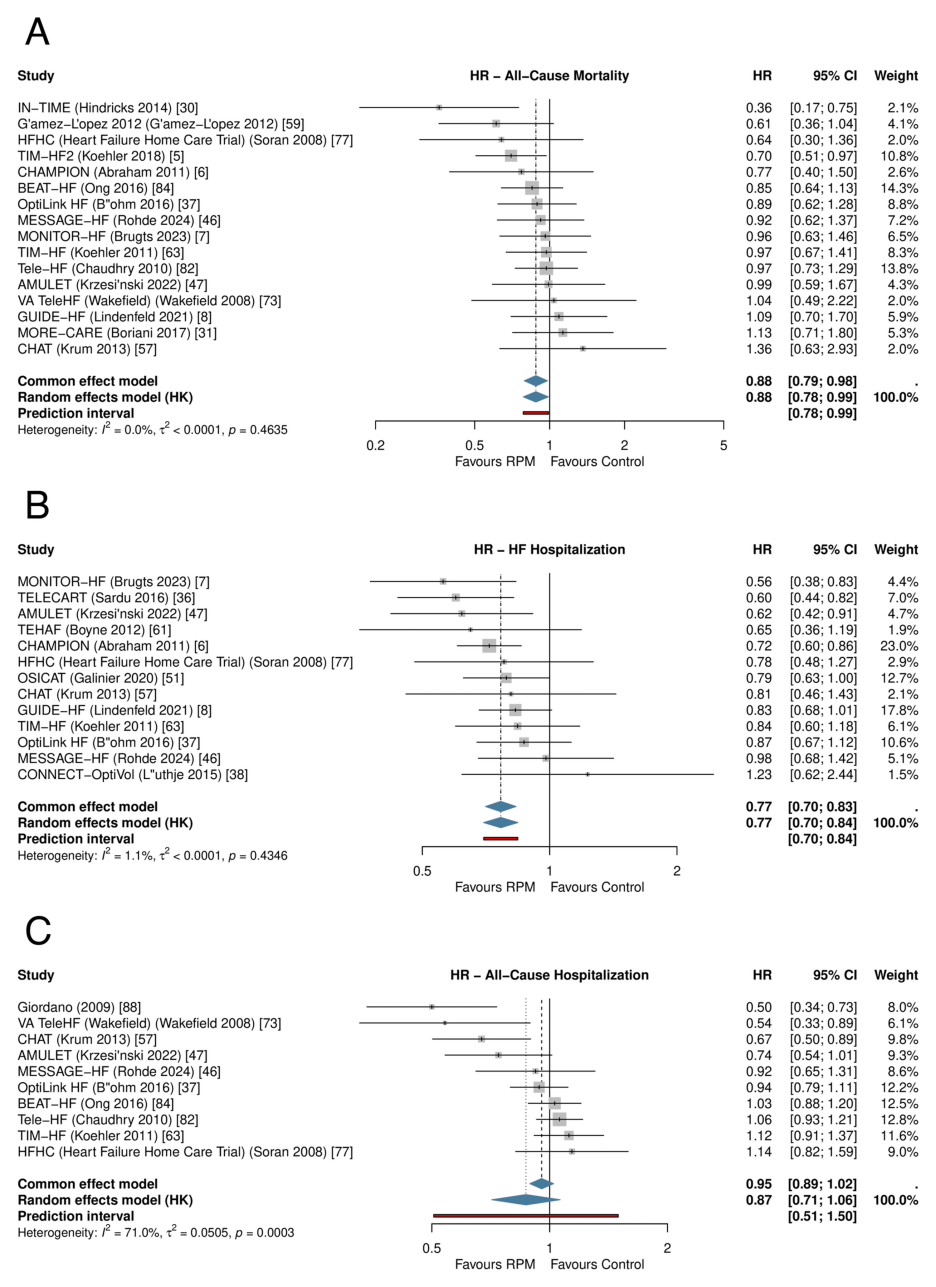
HR sensitivity forests for primary outcomes HR-only sensitivity restricted to studies reporting hazard ratios. (A) All-cause mortality (k=16; HR: 0.880 [0.784, 0.989]). (B) HF hospitalization (k=13; HR: 0.766 [0.698, 0.841]). (C) All-cause hospitalization (k=10; HR 0.870 [0.711, 1.064]). Random-effects meta-analysis with REML and HK adjustment. HR: hazard ratio, REML: restricted maximum likelihood, HK: Hartung-Knapp, CI: confidence interval, HF: heart failure, RPM: remote patient monitoring

RPM significantly reduced all-cause mortality across 41 RCTs (RR: 0.911, 95% CI: 0.842-0.985; P=0.021; I²=0%; τ²=0) (Figure 3, Table 6). The 95% prediction interval was 0.840-0.988, excluding the null under the random-effects model. The number needed to treat was 104 per year. In sensitivity analysis restricted to studies reporting hazard ratios (k=16), the result was consistent (HR: 0.880, 95% CI: 0.784-0.989; P=0.034); HR-based forests for all three primary outcomes are presented in Figure 4.

**Table 6:** Summary of meta-analysis results Headline results table covering primary outcomes (RR, Mantel-Haenszel), HR sensitivity subset, secondary outcomes, and continuous QoL outcomes. Random-effects model (REML variance estimator + Hartung-Knapp-Sidik-Jonkman CI correction). MLHFQ: lower score = better; KCCQ: higher score = better Prediction intervals shown for outcomes with k ≥ 3 studies. RR: risk ratio, HR: hazard ratio, QoL: quality of life, HF: heart failure, CV: cardiovascular, ED: emergency department, MLHFQ: Minnesota Living With Heart Failure Questionnaire, KCCQ: Kansas City Cardiomyopathy Questionnaire, ACM: all-cause mortality, MD: mean difference

Category	Outcome	Measure	k	Effect [95% CI]	P-value	I2 (%)	Prediction Interval
Primary outcomes (RR, Mantel-Haenszel)	All-cause mortality (primary)	RR	41	0.911 [0.842, 0.985]	0.0208	0.0	[0.840, 0.988]
Primary outcomes (RR, Mantel-Haenszel)	HF hospitalization (primary)	RR	39	0.781 [0.710, 0.859]	0.0000	47.2	[0.586, 1.040]
Primary outcomes (RR, Mantel-Haenszel)	All-cause hospitalization (primary)	RR	28	0.959 [0.892, 1.031]	0.2462	54.3	[0.809, 1.137]
Primary sensitivity (HR subset, metagen)	All-cause mortality	HR	16	0.880 [0.784, 0.989]	0.0335	0.0	[0.783, 0.989]
Primary sensitivity (HR subset, metagen)	HF hospitalization	HR	13	0.766 [0.698, 0.841]	0.0000	1.1	[0.698, 0.841]
Primary sensitivity (HR subset, metagen)	All-cause hospitalization	HR	10	0.870 [0.711, 1.064]	0.1514	71.0	[0.505, 1.497]
Secondary outcomes (ratio)	CV mortality	RR	9	0.814 [0.668, 0.992]	0.0433	0.0	-
Secondary outcomes (ratio)	ED visits	RR	6	0.795 [0.550, 1.149]	0.1699	41.4	-
Secondary outcomes (ratio)	Composite ACM + hospitalization	HR	25	0.827 [0.765, 0.894]	0.0000	37.5	-
Secondary outcomes (continuous, MD)	MLHFQ	MD	11	-6.53 [-10.28, -2.79]	0.0030	93.6	-
Secondary outcomes (continuous, MD)	KCCQ	MD	3	8.14 [-24.03, 40.30]	0.3902	83.7	-

Primary Outcomes: HF Hospitalization

**Figure 5:**
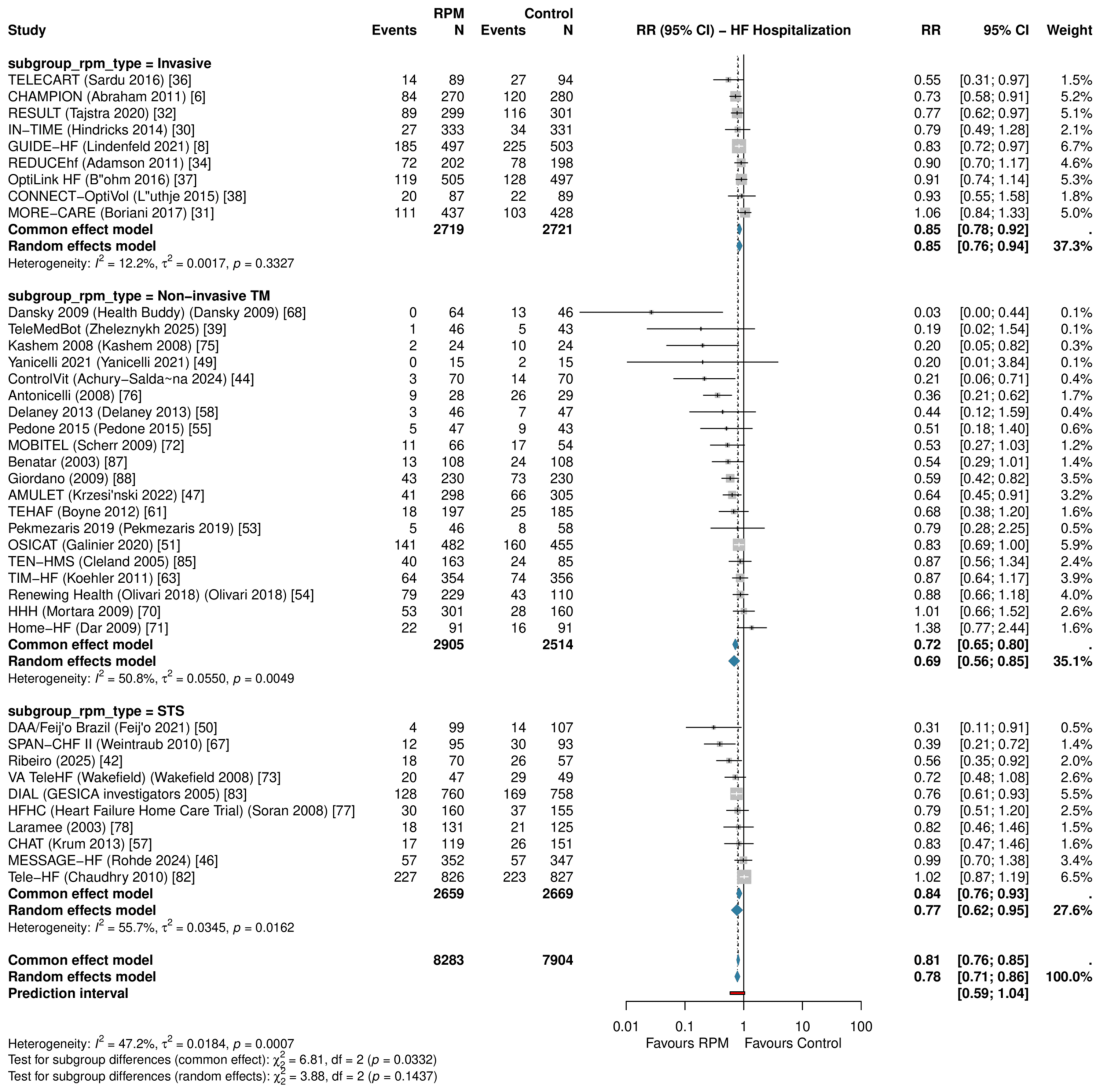
Forest plot of HF hospitalization Pooled RR: 0.781 (95% CI: 0.710-0.859; I²=47.2%; k=39) HF: heart failure, RR: risk ratio, CI: confidence interval, RPM: remote patient monitoring, TM: telemonitoring, STS: structured telephone support

RPM significantly reduced HF hospitalization across 39 RCTs (RR: 0.781, 95% CI: 0.710-0.859; P<0.001; I²=47.2%; τ²=0.018) (Figure 5). The 95% prediction interval was 0.586-1.040, crossing 1.0, consistent with moderate heterogeneity. The NNT was 18 per year. In HR sensitivity (k=13), the effect was more pronounced (HR: 0.766, 95% CI: 0.698-0.841; P<0.001; I²=1.1%; Figure 4B).

Primary Outcomes: All-Cause Hospitalization

**Figure 6:**
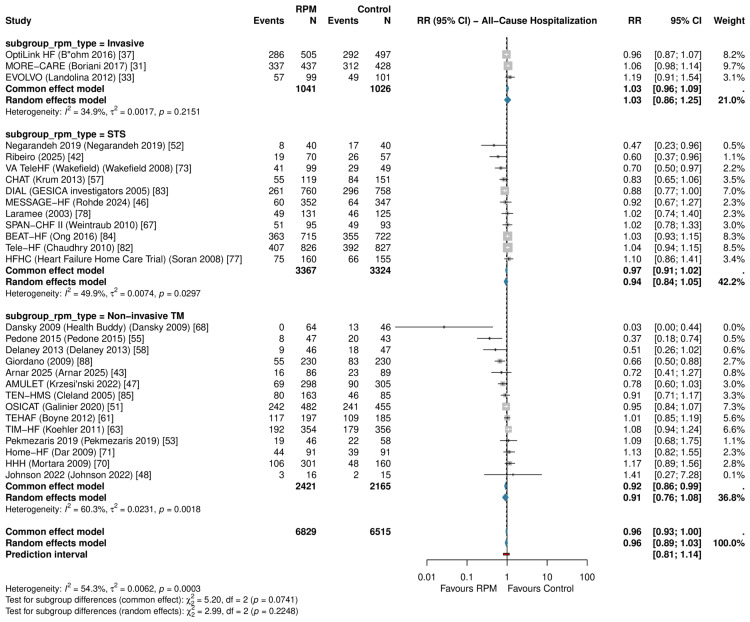
Forest plot of all-cause hospitalization Pooled RR: 0.959 (95% CI: 0.892-1.031; I²=54.3%; k=28) RR: risk ratio, CI: confidence interval, RPM: remote patient monitoring, TM: telemonitoring, STS: structured telephone support

RPM did not significantly reduce all-cause hospitalization across 28 RCTs (RR: 0.959, 95% CI: 0.892-1.031; P=0.246; I²=54.3%) (Figure 6). The prediction interval was 0.809-1.137. The HR sensitivity subset is shown in Figure 4C.

Secondary Outcomes

**Figure 7:**
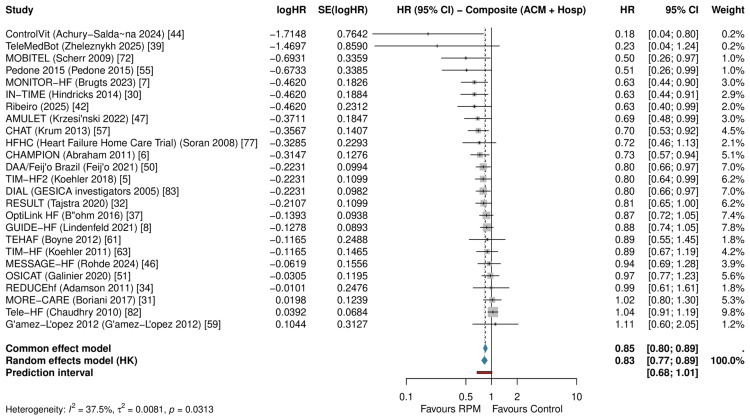
Forest plot of composite all-cause mortality and hospitalization Pooled HR: 0.827 (95% CI: 0.765-0.894; I²=37.5%; k=25) HR: hazard ratio, HK: Hartung-Knapp, CI: confidence interval, RPM: remote patient monitoring, SE: standard error

Cardiovascular mortality was significantly reduced (RR: 0.814, 95% CI: 0.668-0.992; P=0.043; I²=0%; k=9; low certainty after downgrading for imprecision). The composite of all-cause mortality and hospitalization was significantly reduced (HR: 0.827, 95% CI: 0.765-0.894; P<0.001; I²=37.5%; k=25; low certainty after downgrading for indirectness due to mixed effect measures and inconsistency; Figure 7). Emergency department visits showed a non-significant reduction (RR: 0.795, 95% CI: 0.550-1.149; P=0.17; I²=41.4%; k=6). Quality of life measured by MLHFQ improved (MD: -6.53 points, 95% CI: -10.28 to -2.79; P=0.003; I²=93.6%; k=11), favoring RPM (lower scores indicate better quality of life), although the very high heterogeneity makes the pooled mean difference difficult to interpret. KCCQ showed a non-significant improvement (MD: 8.14, 95% CI: -24.03 to 40.30; P=0.39; I²=83.7%; k=3). All secondary effect estimates are summarized together with the primary outcomes in Table 6.

Subgroup Analyses

**Figure 8:**
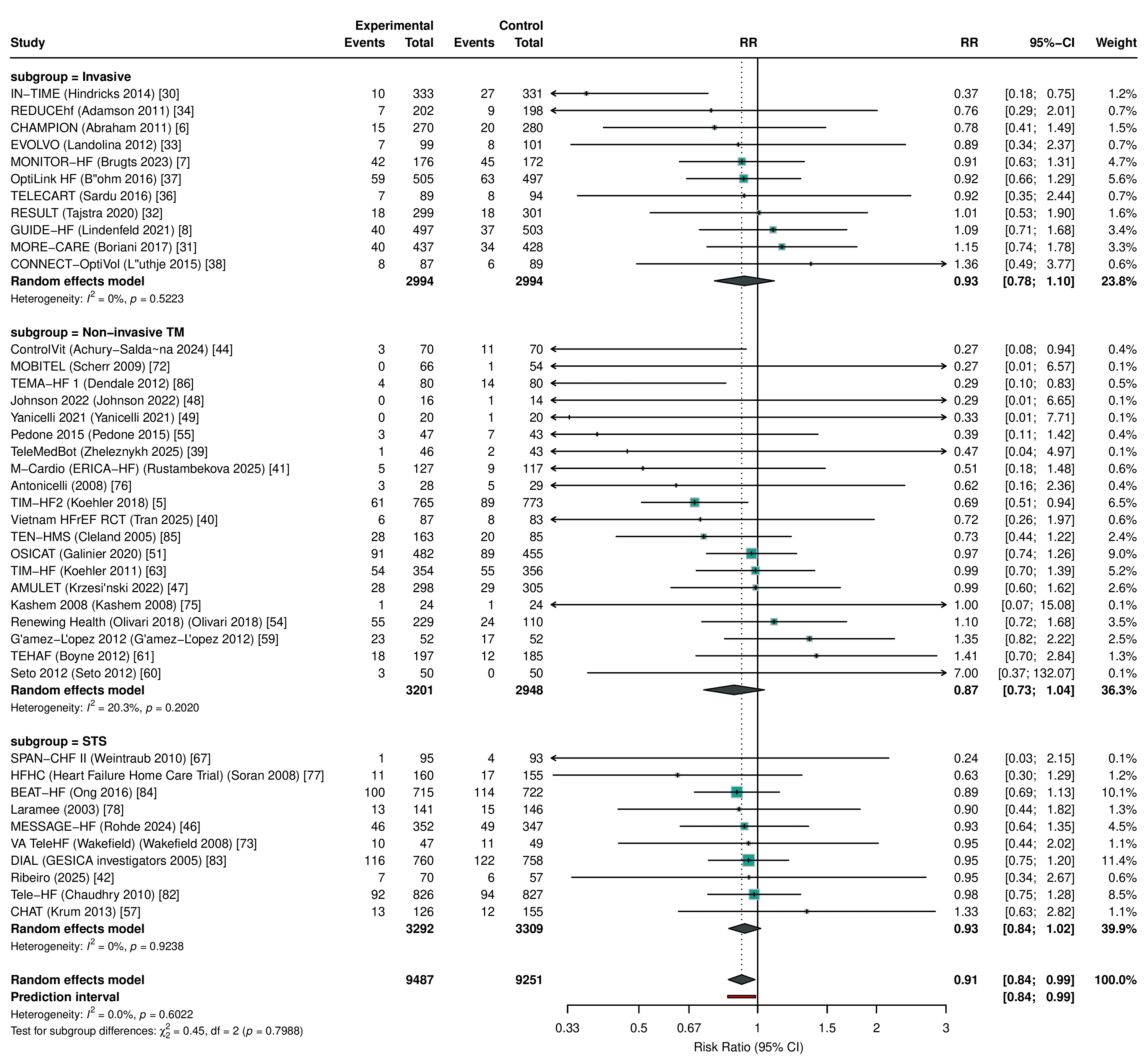
Subgroup analysis of all-cause mortality by RPM type No significant interaction was observed (P interaction = 0.80). RPM: remote patient monitoring, STS: structured telephone support; TM: non-invasive telemonitoring, CI: confidence interval, RR: risk ratio

No statistically significant interaction by RPM type was detected for any primary outcome, although the analysis was likely underpowered to detect moderate subgroup differences given the imbalanced subgroup sizes (STS: k=10, TM k=20, invasive: k=11 for ACM). For all-cause mortality, the test for subgroup differences yielded Pinteraction = 0.80, with similar point estimates across subgroups (non-invasive TM: RR: 0.871; invasive monitoring: RR: 0.925; STS: RR: 0.928; Figure 8). For HF hospitalization, Pinteraction = 0.14, and for all-cause hospitalization, Pinteraction = 0.22.

HF phenotype showed a significant interaction for all-cause hospitalization (Pinteraction = 0.002), with mixed HF phenotype trials showing a directionally favorable estimate (RR: 0.940) compared with HFrEF-only trials (RR: 1.037). This was not significant for all-cause mortality (Pinteraction = 0.46) and was borderline for HF hospitalization (Pinteraction = 0.056). The single trial classified as HFmrEF in the subgroup analysis (M-Cardio (ERICA-HF), mean LVEF: 42%) contributed a directionally favorable but imprecise estimate, although this subgroup comprises only one study and should be interpreted cautiously.

Geographic context was examined as a descriptive subgroup. Among 41 trials reporting all-cause mortality, 13 were classified as rural/remote and 28 as not reported. No significant interaction was observed (Pinteraction = 0.90). The sparse reporting of geographic context (only two trials (TIM-HF2 and CHAT) provided formal rural/urban subgroup analyses) limited the interpretability of this analysis.

**Figure 9:**
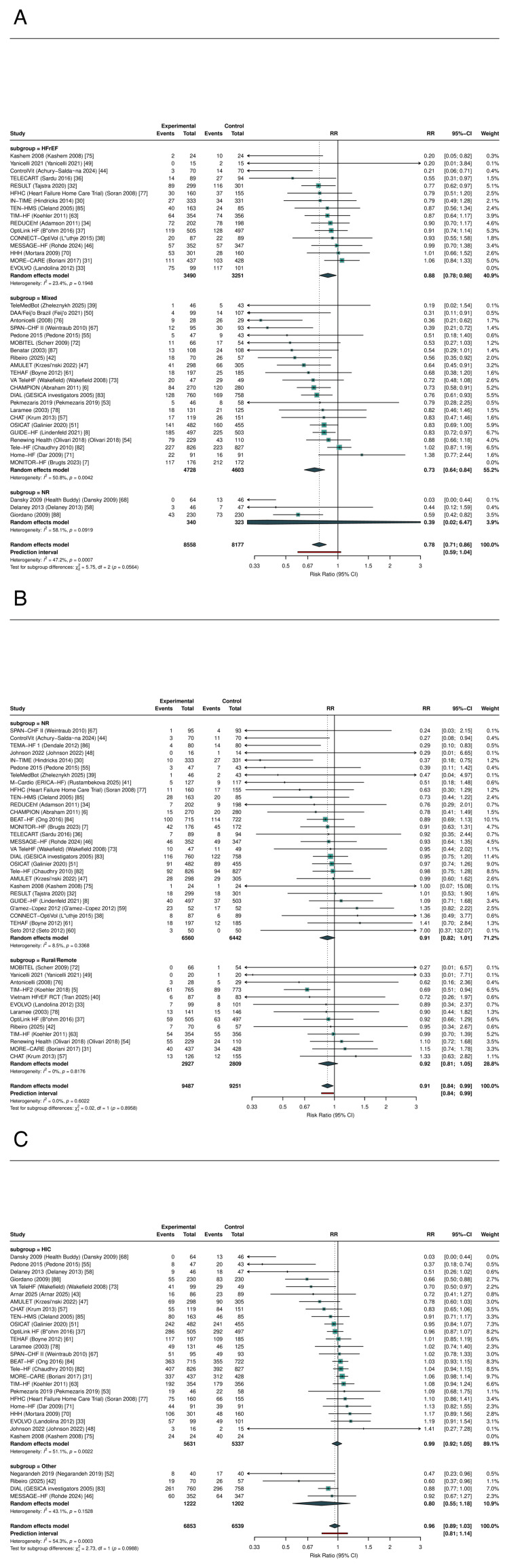
Additional prespecified subgroup analyses (A) HF hospitalization by HF phenotype (P interaction = 0.002). (B) All-cause mortality by geographic context (rural/remote versus not reported; P interaction = 0.90). (C) All-cause hospitalization by country income level (high-income versus other). RR: risk ratio, CI: confidence interval, HFrEF: heart failure with reduced ejection fraction, HF: heart failure, HIC: high-income country

No significant interaction was observed for follow-up duration (≤6 versus >6 months), publication era (≤2015 versus >2015), country income level, or risk of bias for any primary outcome (all Pinteraction > 0.05). Additional subgroup forests covering HF phenotype, geographic context, and country income are shown in Figure 9.

In multivariable meta-regression for HF hospitalization, the total sample size was borderline rather than statistically significant after excluding recurrent-count rows from the binary RR analysis (β=0.00026, P=0.063). Mean LVEF was associated with treatment effect (β=-0.032, P=0.0049), but this ecological finding should be interpreted cautiously because study-level covariates can be confounded by intervention type, era, and patient selection. No covariates were significant predictors for all-cause mortality.

Sensitivity Analyses

**Figure 10:**
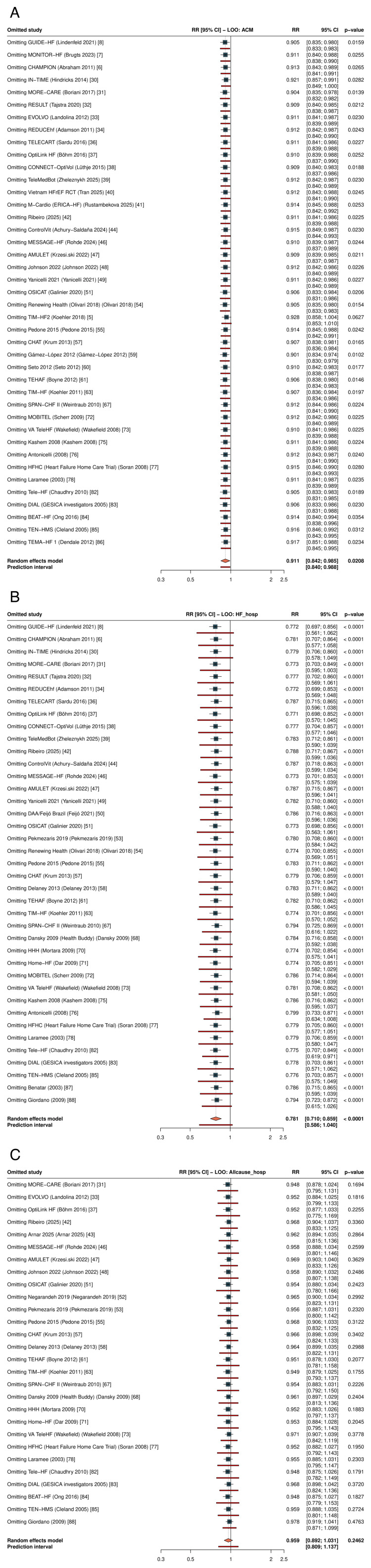
Leave-one-out sensitivity analysis Pooled RR after omitting each study from the meta-analysis. (A) All-cause mortality: range [0.901, 0.928] across all omissions. (B) HF hospitalization. (C) All-cause hospitalization. The full pooled estimate (diamond) is shown for reference at the bottom. RR: risk ratio, CI: confidence interval, HF: heart failure

All 10 prespecified sensitivity analyses were directionally consistent with the primary results for both all-cause mortality and HF hospitalization (Table 7). For all-cause mortality, leave-one-out analysis showed pooled RR ranging from 0.901 to 0.928, with no single study disproportionately influencing the result (Figure 10A). The fixed-effect model was consistent (RR: 0.900, P=0.009). Excluding invasive RPM studies yielded an RR of 0.906 (P=0.038), excluding high RoB studies yielded an RR of 0.914 (P=0.031), and restricting to large trials (N≥200) yielded an RR of 0.918 (P=0.033; k=24). For HF hospitalization, all sensitivities remained statistically significant, including the large-trial sensitivity (RR: 0.837, 95% CI: 0.779-0.899; k=23) and leave-one-out (Figure 10B); leave-one-out for all-cause hospitalization is shown in Figure 10C.

**Table 7:** Sensitivity analyses for primary outcomes RR: risk ratio, HKSJ: Hartung-Knapp-Sidik-Jonkman, RPM: remote patient monitoring, RCT: randomized controlled trial, RoB: risk of bias, ACM: all-cause mortality

Outcome	Analysis	k	RR	RR range (lower)	RR range (upper)	P-value	I² (%)	τ²	Note	95% CI (lower)	95% CI (upper)
ACM	10_large_trials_n200	24	0.918	NA	NA	0.0331	0	0	N>=200 only (n excl.=17)	0.849	0.993
ACM	11_incl_active_comparators	42	0.918	NA	NA	0.0372	0	0	Post-hoc: including MIGHTY-HEART active comparator (CO-0570)	0.848	0.995
ACM	1_leave_one_out	40	NA	0.901	0.928	NA	NA	NA	LOO pooled-RR range [0.901-0.928] across k=41 iterations	NA	NA
ACM	2_fixed_effect	41	0.9	NA	NA	0.0087	0	NA	Fixed-effect (common); no HKSJ correction	0.832	0.974
ACM	3_usual_care_only	37	0.91	NA	NA	0.023	0	0	Comparator type = usual care only (n excl.=4)	0.839	0.986
ACM	4_excl_invasive	30	0.906	NA	NA	0.0382	0	0	Excl. Invasive RPM (n excl.=11)	0.826	0.994
ACM	5_excl_cluster_rct	40	0.907	NA	NA	0.0165	0	0	Excl. CHAT (CO-0944) cluster RCT	0.838	0.981
ACM	6_low_rob_only	35	0.914	NA	NA	0.0314	0	0	Excl. High RoB (n excl.=6)	0.843	0.992
ACM	7_excl_outliers	36	0.93	NA	NA	0.043	0	0	Excl. flagged outliers (n excl.=5)	0.868	0.998
ACM	8_hr_only_subset	16	0.88	NA	NA	0.0335	0	0	HR-reporting studies only (metagen); k=16	0.784	0.989
ACM	9_fu_ge3mo	41	0.911	NA	NA	0.0208	0	0	FU >=3 months only (n excl.=0)	0.842	0.985
Allcause_hosp	10_large_trials_n200	17	0.988	NA	NA	0.6702	39.4	0.0025	N>=200 only (n excl.=12)	0.934	1.046
Allcause_hosp	11_incl_active_comparators	29	0.965	NA	NA	0.2765	52.7	0.0046	Post-hoc: including MIGHTY-HEART active comparator (CO-0570); excluded recurrent-count rows incompatible with binary RR (n=1)	0.903	1.031
Allcause_hosp	1_leave_one_out	27	NA	0.948	0.978	NA	NA	NA	LOO pooled-RR range [0.948–0.978] across k=28 iterations	NA	NA
Allcause_hosp	2_fixed_effect	28	0.964	NA	NA	0.048	54.3	NA	Fixed-effect (common); no HKSJ correction	0.93	1
Allcause_hosp	3_usual_care_only	23	0.92	NA	NA	0.0745	57.1	0.0102	Comparator type = usual care only (n excl.=5); excluded recurrent-count rows incompatible with binary RR (n=1)	0.838	1.009
Allcause_hosp	4_excl_invasive	25	0.931	NA	NA	0.1098	54.6	0.01	Excl. Invasive RPM (n excl.=3); excluded recurrent-count rows incompatible with binary RR (n=1)	0.851	1.018
Allcause_hosp	5_excl_cluster_rct	27	0.966	NA	NA	0.3402	54.5	0.0053	Excl. CHAT (CO-0944) cluster RCT; excluded recurrent-count rows incompatible with binary RR (n=1)	0.898	1.039
Allcause_hosp	6_low_rob_only	23	0.98	NA	NA	0.4627	39	0.0023	Excl. High RoB (n excl.=5); excluded recurrent-count rows incompatible with binary RR (n=1)	0.927	1.036
Allcause_hosp	7_excl_outliers	26	0.969	NA	NA	0.3031	44.7	0.0045	Excl. flagged outliers (n excl.=3)	0.911	1.031
Allcause_hosp	8_hr_only_subset	10	0.87	NA	NA	0.1514	71	0.0505	HR-reporting studies only (metagen); k=10	0.711	1.064
Allcause_hosp	9_fu_ge3mo	27	0.965	NA	NA	0.2992	52.8	0.0053	FU >=3 months only (n excl.=1); excluded recurrent-count rows incompatible with binary RR (n=1)	0.9	1.034
HF_hosp	10_large_trials_n200	23	0.837	NA	NA	0	21.8	0.0059	N>=200 only (n excl.=16); excluded recurrent-count rows incompatible with binary RR (n=2)	0.779	0.899
HF_hosp	11_incl_active_comparators	40	0.79	NA	NA	0	46.7	0.0164	Post-hoc: including MIGHTY-HEART active comparator (CO-0570); excluded recurrent-count rows incompatible with binary RR (n=2)	0.721	0.865
HF_hosp	1_leave_one_out	38	NA	0.771	0.799	NA	NA	NA	LOO pooled-RR range [0.771–0.799] across k=39 iterations	NA	NA
HF_hosp	2_fixed_effect	39	0.805	NA	NA	0	47.2	NA	Fixed-effect (common); no HKSJ correction	0.763	0.85
HF_hosp	3_usual_care_only	33	0.793	NA	NA	0	43.7	0.0108	Comparator type = usual care only (n excl.=7); excluded recurrent-count rows incompatible with binary RR (n=1)	0.723	0.87
HF_hosp	4_excl_invasive	30	0.725	NA	NA	1E-04	53	0.0399	Excl. Invasive RPM (n excl.=11)	0.629	0.835
HF_hosp	5_excl_cluster_rct	38	0.779	NA	NA	0	48.6	0.0197	Excl. CHAT (CO-0944) cluster RCT; excluded recurrent-count rows incompatible with binary RR (n=2)	0.706	0.859
HF_hosp	6_low_rob_only	31	0.797	NA	NA	0	46.3	0.0143	Excl. High RoB (n excl.=8); excluded recurrent-count rows incompatible with binary RR (n=2)	0.724	0.877
HF_hosp	7_excl_outliers	33	0.812	NA	NA	0	33.3	0.0102	Excl. flagged outliers (n excl.=6); excluded recurrent-count rows incompatible with binary RR (n=2)	0.75	0.878
HF_hosp	8_hr_only_subset	13	0.766	NA	NA	0	1.1	0	HR-reporting studies only (metagen); k=13	0.698	0.841
HF_hosp	9_fu_ge3mo	39	0.781	NA	NA	0	47.2	0.0184	FU >=3 months only (n excl.=0); excluded recurrent-count rows incompatible with binary RR (n=2)	0.71	0.859

Publication Bias

**Figure 11:**
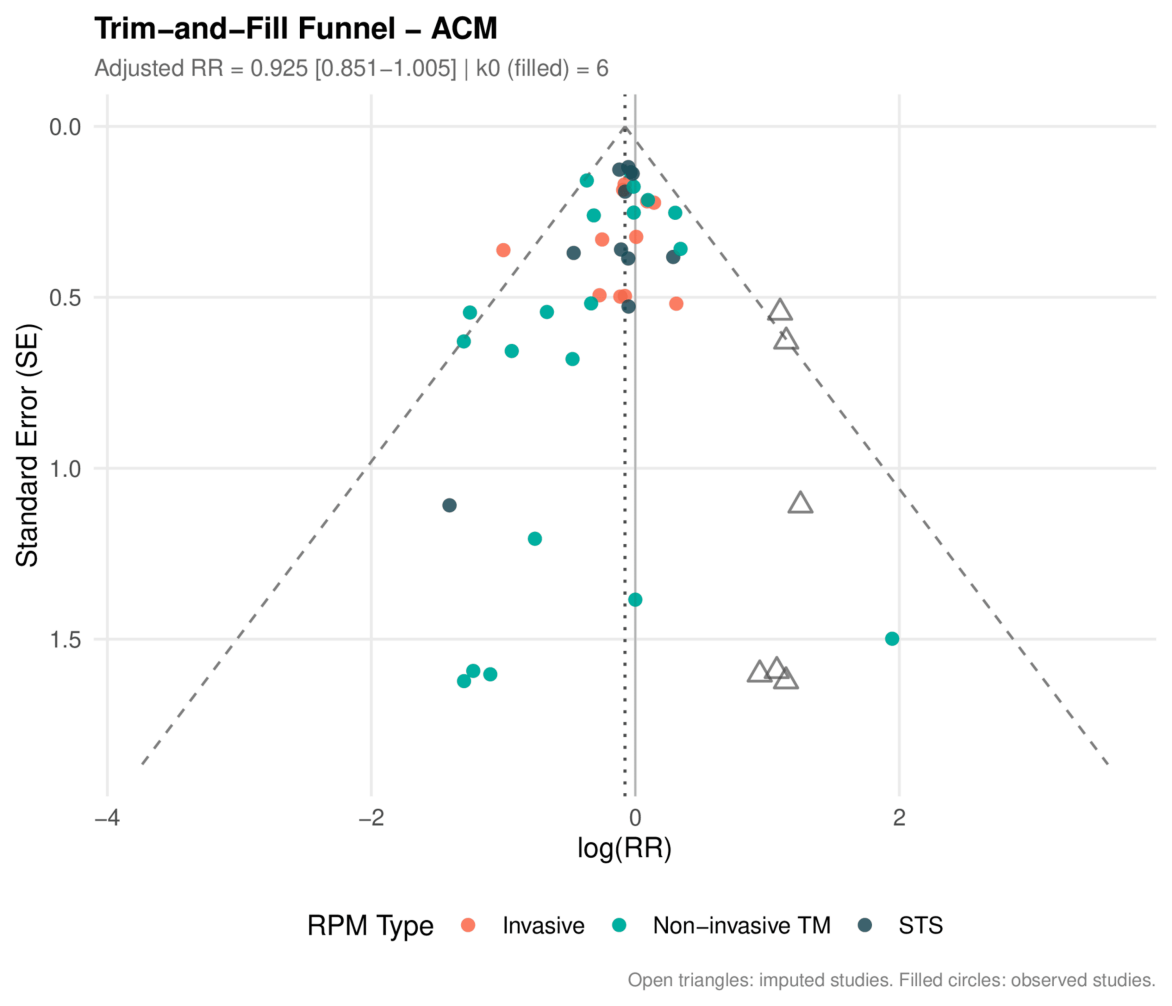
Funnel plot with trim-and-fill analysis for all-cause mortality Open circles represent observed studies; filled circles represent imputed studies (k₀=6). Adjusted RR: 0.925 (95% CI: 0.851-1.005) RR: risk ratio, CI: confidence interval, RPM: remote patient monitoring, TM: telemonitoring, STS: structured telephone support

**Figure 12:**
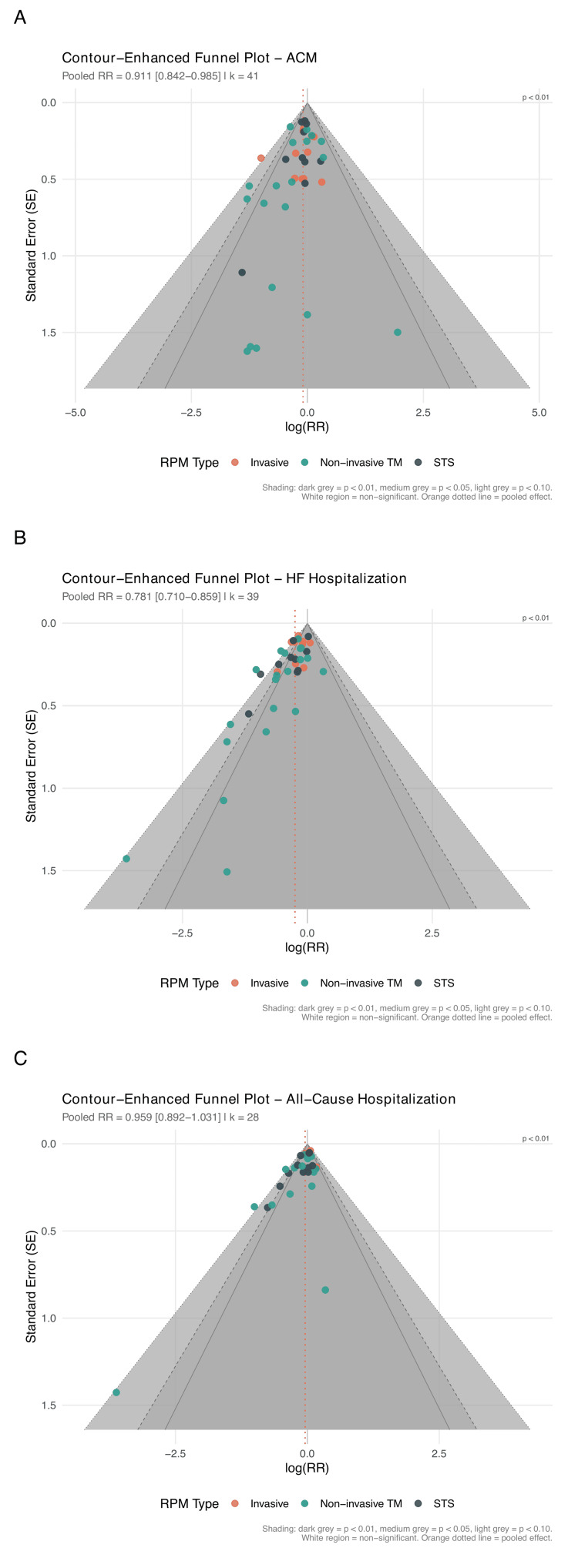
Contour-enhanced funnel plots for the three primary binary outcomes Shaded regions indicate p<0.10, p<0.05, and p<0.01 thresholds. (A) All-cause mortality. (B) HF hospitalization. (C) All-cause hospitalization. RPM technology indicated by color. RR: risk ratio, HF: heart failure, RPM: remote patient monitoring, TM: telemonitoring, STS: structured telephone support

For all-cause mortality, Egger's test was significant (P=0.040) and Begg's test was significant (P=0.014), suggesting funnel plot asymmetry. Peters' regression test was non-significant (P=0.246), and the large-trial sensitivity analysis (N≥200; k=24) yielded an RR of 0.918 (P=0.033), indicating that the mortality reduction persisted when small studies were excluded. Trim-and-fill analysis estimated six missing studies on the right side and yielded an adjusted RR of 0.925 (95% CI: 0.851-1.005; P=0.064), attenuating the mortality estimate to borderline non-significance (Figure 11). The contour-enhanced funnel plot (Figure 12A) suggested that asymmetry was driven primarily by small studies in regions of statistical significance, consistent with small-study effects; selective non-publication cannot be excluded.

For HF hospitalization, all three tests were significant (Egger: P<0.001, Begg: P<0.001, Peters: P=0.0002), with trim-and-fill estimating 12 missing studies and an adjusted RR of 0.830 (95% CI: 0.737-0.934; P=0.003). The substantial asymmetry, also shown in Figure 12B, was consistent with small-study effects and potentially greater treatment effects in smaller, more intensive interventions. The funnel plot for all-cause hospitalization is shown in Figure 12C.

Trial Sequential Analysis

**Figure 13:**
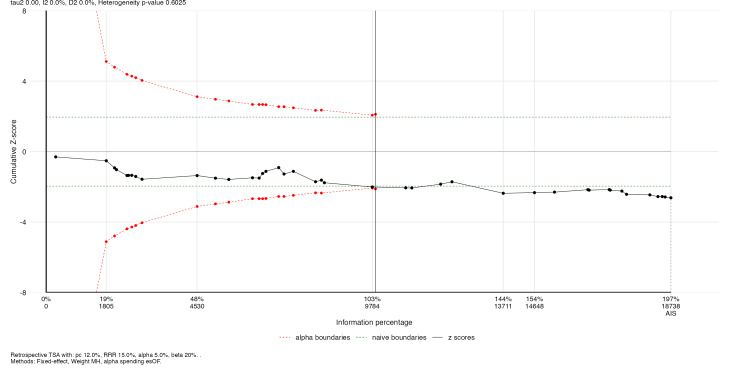
Trial sequential analysis for all-cause mortality (RRR=15%) α=0.05, β=0.20, O’Brien-Fleming boundaries The cumulative Z-curve crosses the monitoring boundary, and the accrued information size (18,738) exceeds the sequentially adjusted required information size (9,880), supporting a stable mortality benefit under this assumption. RRR: relative risk reduction, AIS: accrued information size, TSA: trial sequential analysis

**Figure 14:**
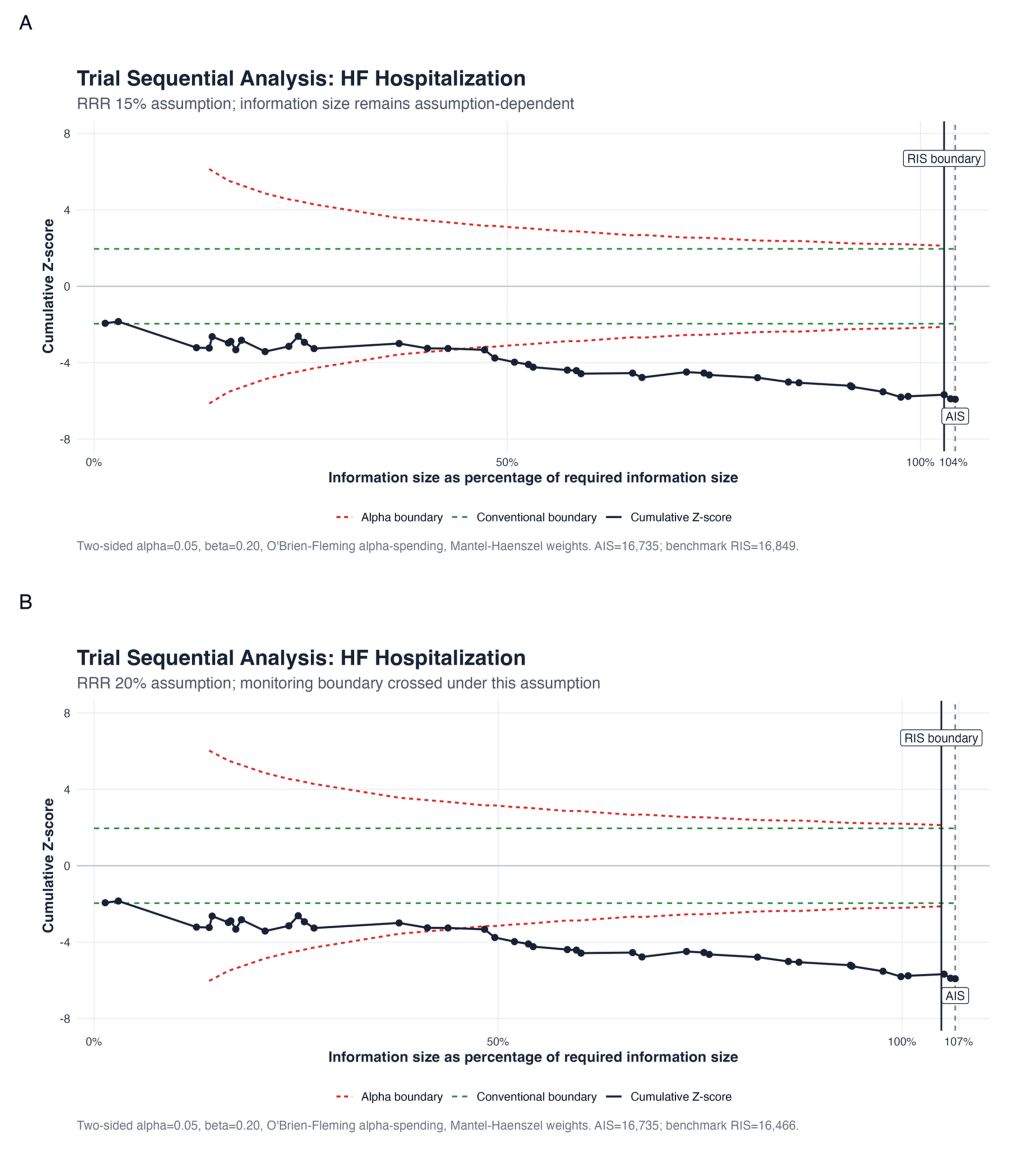
Trial sequential analysis for HF hospitalization (A) RRR=15%: accrued information size below sequentially adjusted required size. (B) RRR=20%: cumulative Z-curve crosses the monitoring boundary, indicating sufficient evidence at this effect-size assumption. HF: heart failure, RRR: relative risk reduction, AIS: accrued information size

For all-cause mortality with RRR=15%, the accrued information size (AIS=18,738 patients) exceeded the sequentially adjusted required information size (9,880 patients), and the cumulative Z-curve crossed the O'Brien-Fleming monitoring boundary, supporting a stable mortality benefit under this assumption (Figure 13). For HF hospitalization, the AIS did not reach the heterogeneity-adjusted benchmark at RRR=15%, but crossed the monitoring boundary at RRR=20%, indicating that the TSA result is assumption-dependent (Figure 14).

NNT and GRADE

**Figure 15:**
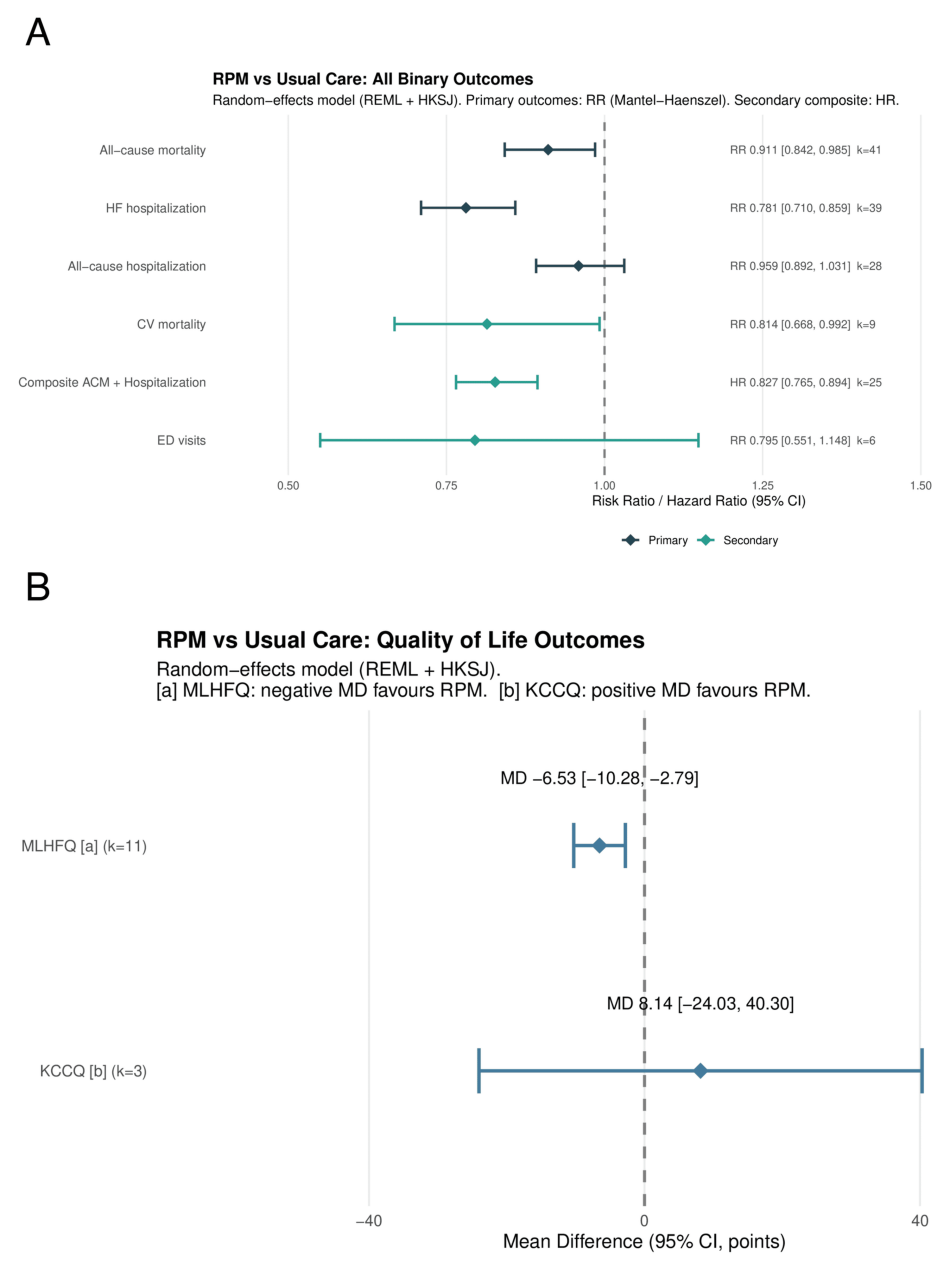
Summary forest plots of all pooled outcomes (A) Binary outcomes (RR/HR with 95% CI), including primary and secondary measures. (B) Continuous quality-of-life outcomes (MLHFQ and KCCQ) reported as mean differences. Random-effects model (REML + HKSJ) RR: risk ratio, CI: confidence interval, REML: restricted maximum likelihood, HKSJ: Hartung-Knapp-Sidik-Jonkman, MLHFQ: Minnesota Living With Heart Failure Questionnaire, KCCQ: Kansas City Cardiomyopathy Questionnaire, HR: hazard ratio, HF: heart failure, CV: cardiovascular, ED: emergency department, RPM: remote patient monitoring, MD: mean difference

For all-cause mortality, the NNT was 93 overall and 104 per year (based on a control event rate of 12% over a median follow-up of 13.5 months among studies contributing valid participant-level event counts). For HF hospitalization, the NNT was 17 overall and 18 per year (control event rate: 26.9%, median follow-up: 13.2 months), after excluding two recurrent-count rows from CER/NNT calculation. NNT annualization assumes a constant hazard rate, which may overestimate annual benefit for outcomes with non-proportional hazards; these estimates should be interpreted with this caveat. Furthermore, the prediction interval for HF hospitalization crosses 1.0, meaning that in some clinical settings, the NNT could be substantially larger or even infinite. GRADE certainty was moderate for all-cause mortality (downgraded for suspected publication bias), low for HF hospitalization (downgraded for inconsistency and suspected publication bias), very low for all-cause hospitalization, low for cardiovascular mortality (downgraded for very serious imprecision: borderline significance, small k=9, optimal information size not met), and low for the composite outcome (downgraded for indirectness due to mixed effect measures and inconsistency). The full GRADE evidence profile is provided in Table 8, and a summary of all binary and continuous outcomes is shown in Figure 15. Safety and adherence outcomes per study are shown in Table 9.

**Table 8:** GRADE evidence profile across primary and secondary outcomes GRADE: Grading of Recommendations, Assessment, Development, and Evaluations, HF: heart failure, CV: cardiovascular, MLHFQ: Minnesota Living With Heart Failure Questionnaire, KCCQ: Kansas City Cardiomyopathy Questionnaire, ED: emergency department, ACM: all-cause mortality

Outcome	Studies (N)	Risk of bias	Inconsistency	Indirectness	Imprecision	Publication bias	Certainty
All-cause mortality	41	Not serious	Not serious (I2=0%)	Not serious	Not serious	Suspected	Moderate
HF hospitalization	39	Not serious	Serious (I2=47%)	Not serious	Not serious	Suspected	Low
All-cause hospitalization	28	Not serious	Serious (I2=54%)	Not serious	Serious	Suspected	Very low
CV mortality	9	Not serious	Not serious (I2=0%)	Not serious	Very serious	Undetected	Low
Composite ACM + hospitalization	25	Not serious	Serious (I2=38%)	Serious	Not serious	Undetected	Low
MLHFQ	11	Not serious	Very serious (I2=94%)	Not serious	Not serious	Undetected	Low
ED visits	6	Not serious	Serious (I2=41%)	Not serious	Serious	Undetected	Low
KCCQ	3	Serious	Very serious (I2=84%)	Not serious	Serious	Undetected	Very low

**Table 9:** Safety and adherence outcomes (per study) RPM: remote patient monitoring, STS: structured telephone support, TM: telemonitoring, IQR: interquartile range

RPM category	Studies (k)	Mean (%)	Median (%)	IQR (%)	Range (%)
STS	8	73.5	70.3	63.2-83.5	55-97
Non-invasive TM	21	80	83	76-90	41-97
Invasive	7	83.8	85	83.4-85.5	76-88
Overall	36	79.3	83	74.5-88.2	41-97

Interpretation

This systematic review and meta-analysis, among the most comprehensive to date on RPM in HF, encompassing 59 poolable RCTs, demonstrates that RPM significantly reduces all-cause mortality by 9% (RR: 0.911; NNT: 104 per year) and HF hospitalization by 22% (RR: 0.781; NNT: 18 per year), with no significant effect on all-cause hospitalization. These findings were directionally robust across 10 sensitivity analyses, while publication-bias and small-study signals require cautious interpretation of the headline effects.

The mortality reduction is statistically consistent (I²=0%), with a narrow prediction interval (0.840-0.988). This statistical homogeneity should not be overinterpreted as clinical interchangeability across RPM modalities, eras, and healthcare systems. The HKSJ method provides more reliable confidence interval coverage than the DerSimonian-Laird approach, particularly with a moderate number of studies [20,21].

Although no statistically significant interaction by RPM technology type was detected, the analysis had limited power to detect moderate differences given the subgroup sample sizes. Ezimoha et al. reported a significant RPM-type interaction (P=0.007) in a smaller analysis (k=15) [16]. The non-significant interaction in our larger analysis should not be interpreted as proof of equivalence among technologies. Nonetheless, the directional consistency across all three RPM categories suggests that the mechanism of benefit may relate less to any single device and more to the clinical feedback loop that RPM enables: the systematic detection of deterioration, timely clinical response, and guided self-management. This interpretation aligns with the TIM-HF2 rationale, where the structured management protocol was considered as important as the monitoring technology [5].

Our results update and extend the Cochrane reviews by Inglis et al., which reported STS mortality RR of 0.87 (95% CI: 0.77-0.98) and TM mortality RR of 0.80 (95% CI: 0.68-0.94) in 2015 [4]. Our overall RR of 0.911 is broadly consistent with but less favorable than these earlier estimates, reflecting the inclusion of several large neutral trials published since 2015 (BEAT-HF, OSICAT, PRADOC, and MESSAGE-HF) and the dilution of earlier positive signals by more pragmatic, larger-scale designs. With an NNT of 104 per year for mortality and 18 per year for HF hospitalization, the absolute benefit remains potentially meaningful, but the HF hospitalization estimate should be interpreted alongside the large-trial sensitivity and publication-bias analyses.

Three contemporary meta-analyses published in 2025 provide relevant context. De Lathauwer et al. pooled 41 RCTs (16,312 patients) with access to Embase and reported an OR of 0.81 (95% CI: 0.69-0.95) for mortality, directionally consistent with our RR of 0.911 [14]. Their component analysis identified video communication as an effectiveness enhancer, a finding our data could not evaluate, but that warrants future investigation. Dobre et al. reported 105 studies (45,072 patients) across multiple databases [15]; however, their substantially larger yield reflects inclusion of non-randomized designs and post-hoc analyses, whereas our review was restricted to RCTs with extractable outcome data. Ezimoha et al. (15 RCTs) reported a significant RPM-type interaction for mortality (P=0.007) [16], contrasting with our non-significant interaction; however, their smaller sample (k=15 versus our k=41) and different subgroup definitions limit direct comparison. The concordance of the mortality benefit direction across independent reviews strengthens confidence in the overall signal, although the exact effect size remains sensitive to analytic choices and small-study effects.

The invasive monitoring evidence deserves specific commentary. The CHAMPION trial showed a striking 28% reduction in HF hospitalization with CardioMEMS-guided management [6], subsequently replicated in MONITOR-HF (HR: 0.56) [7]. However, the GUIDE-HF trial yielded neutral overall results (HR: 0.88, P=0.16), with benefit confined to a pre-COVID sensitivity analysis [8]: the pandemic reduced event rates in both arms, narrowing the between-group difference. The MORE-CARE trial, which evaluated a different mechanism (remote monitoring of cardiac resynchronization therapy defibrillator (CRT-D) device diagnostics rather than direct hemodynamic measurement), was similarly neutral. When pooled, invasive monitoring did not significantly reduce all-cause mortality (RR: 0.925, P=0.35), although HF hospitalization was significantly reduced (RR: 0.846, P=0.006). This pattern is consistent with the mechanism of action: hemodynamic monitoring primarily targets congestion (the driver of hospitalization) rather than arrhythmia or pump failure (drivers of mortality).

IN-TIME warrants specific discussion as a potential outlier. This trial of Biotronik Home Monitoring using implantable cardioverter-defibrillator (ICD) and CRT-D devices reported an extreme mortality hazard ratio of 0.36 (10 versus 27 deaths; n=664) [30]. Leave-one-out analysis demonstrated that removing any single trial shifted the pooled all-cause mortality RR within a narrow range (0.901-0.928), confirming that no single trial, including IN-TIME, disproportionately drives the overall mortality conclusion.

HF phenotype showed limited interaction effects. The predominance of HFrEF or mixed populations in the included trials reflects the historical focus of RPM research. None of the 59 poolable trials enrolled patients with HFpEF exclusively; PROACTIVE-HF, which targeted HFpEF, was excluded from quantitative synthesis owing to its feasibility design without a usual-care control arm for the primary endpoint [9]. The significant interaction for all-cause hospitalization by HF phenotype (P=0.002) should be interpreted cautiously, given the small number of subgroup-specific trials and the potential for confounding by intervention type and era.

Geographic access was examined as a secondary descriptive analysis, motivated by the expectation that RPM could provide the greatest incremental benefit in populations with limited access to in-person HF care [3]. The findings were informative primarily as a reporting gap. Only 2 of 59 poolable trials reported formal rural/urban subgroup analyses: TIM-HF2 found no significant interaction (P=0.66), and CHAT was specifically designed for rural and remote patients. Laramee et al. (2003) reported substantial differences between rural (2% HF rehospitalization) and urban (14%) subgroups, but this was a single-center study [78]. The overwhelming majority of trials did not report geographic context at all, making patient-level geographic effect modification infeasible. If RPM is to be deployed as a strategy to address healthcare disparities, trials must be designed and powered to detect geographic access interactions.

The publication bias findings warrant cautious interpretation. Both Egger's (P=0.040) and Begg's (P=0.014) tests were significant for all-cause mortality, while Peters' regression test was non-significant (P=0.246). Trim-and-fill analysis imputed six studies, yielding an adjusted RR of 0.925 (95% CI: 0.851-1.005; P=0.064), attenuating the mortality estimate to borderline non-significance. The large-trial sensitivity analysis (N≥200; k=24) yielded an RR of 0.918 (P=0.033), demonstrating that the mortality signal persists when small studies are excluded, although with a smaller effect size. The contour-enhanced funnel plots suggest that asymmetry is driven primarily by small studies in significance regions, consistent with small-study effects, but selective non-publication cannot be excluded. For HF hospitalization, the publication bias signal was more pronounced: all three tests were significant (Egger: P<0.001, Begg: P<0.001, Peters: P=0.0002), and trim-and-fill imputed 12 studies (31% of k=39), yielding an adjusted RR of 0.830. This degree of asymmetry warrants caution in interpreting the HF hospitalization estimate.

The large-trial sensitivity complements the publication bias analysis and suggests that the HF hospitalization benefit may be closer to the attenuated large-trial estimate (RR: 0.837, sensitivity #10) than the overall pooled estimate (RR: 0.781) in pragmatic settings. In multivariable meta-regression, the total sample size was borderline (P=0.063) rather than statistically significant after excluding recurrent-count rows from binary pooling. No covariates were significant predictors for all-cause mortality, consistent with the low statistical heterogeneity (I²=0%) observed for this outcome.

Strengths

This review has several strengths. It is among the most comprehensive RPM meta-analyses of RCTs, including 59 poolable trials across all three RPM categories. The search encompassed four databases without language restrictions. The analytic approach used REML+HKSJ, included 10 sensitivity analyses per outcome, applied TSA as a sequential-monitoring sensitivity framework, and used GRADE to rate certainty. The geographic access analysis highlights an important reporting gap.

Limitations

Several limitations should be acknowledged. First, the open-label design inherent to RPM trials introduces potential performance and detection bias, although hard endpoints (mortality and hospitalization) are less susceptible to this bias than patient-reported outcomes. Second, heterogeneity was moderate for HF hospitalization (I²=47.2%) and all-cause hospitalization (I²=54.3%), likely reflecting variation in intervention intensity, healthcare system context, and patient populations across decades of research. Third, geographic access data were sparse, limiting the interpretation of this secondary descriptive analysis. Fourth, some older trials had small sample sizes, contributing to the small-study effects detected in publication bias analysis. Fifth, the mix of effect measures (RR from event counts and HR from time-to-event analyses) required parallel sensitivity analyses rather than a single unified model; additionally, recurrent-event counts could not be pooled as binary participant-level risks and were excluded from RR/NNT calculations. Sixth, the median follow-up of six months may not capture the longer-term effects of sustained RPM programs. Seventh, the search was limited to four databases (PubMed/MEDLINE, Cochrane CENTRAL, ClinicalTrials.gov, and WHO ICTRP); Embase and CINAHL were not searched due to the absence of institutional access, a recognized deviation from Cochrane Handbook recommendations [19]. However, several factors mitigate the potential impact of this omission. Cochrane CENTRAL partially aggregates Embase-indexed content: 42.9% of the 2,138 CENTRAL records retrieved in our search carried an Embase identifier, and Sampson et al. found that CENTRAL captures a growing proportion of Embase RCTs [13]. Slobogean et al. demonstrated that PubMed combined with CENTRAL identifies approximately 97% of relevant RCTs in therapeutic systematic reviews [12]. Of our 65 included studies, 63 (96.9%) had PubMed identifiers, suggesting minimal unique Embase-only yield for this topic. Furthermore, cross-referencing our study list against three contemporary meta-analyses that searched Embase (De Lathauwer et al. [14], 41 RCTs; Dobre et al. [15], 105 studies; and Ezimoha et al. [16], 15 RCTs) [14-16] revealed concordant mortality direction across reviews (Appendix A). Nevertheless, the possibility that Embase-only European or Asian RCTs were missed cannot be excluded, and this limitation is reflected in the GRADE publication bias domain assessment. Eighth, screening used semi-automated rules with manual adjudication and audit, and archived files do not preserve complete independent reviewer-level decisions. Ninth, although extraction was completed using a two-reviewer consensus process, the archived dataset retains final consensus values rather than a complete row-level history of all initial reviewer disagreements.

Clinical Implications

The NNT of 18 per year for HF hospitalization, while not directly comparable due to differences in patient populations, outcome definitions, and follow-up durations, is in a broadly similar range to NNTs reported for sodium-glucose cotransporter-2 (SGLT2) inhibitors in DAPA-HF and EMPEROR-Reduced (~19-21 per year for the composite of cardiovascular (CV) death and HF hospitalization) and mineralocorticoid receptor antagonists in EMPHASIS-HF (~22 per year). Combined with the mortality benefit (NNT of 104 per year), these results support considering RPM programs as an adjunct to guideline-directed medical therapy in HF, particularly where clinical response pathways are well defined. The absence of statistically significant differential effect by RPM type, while acknowledging the limited power to detect moderate differences, suggests that healthcare systems can consider selecting the technology most appropriate to their resources and infrastructure, an important consideration for low- and middle-income countries and for rural health systems where invasive monitoring may not be feasible.

Future Research

Future research should prioritize geographic access-enriched trials to determine whether RPM differentially benefits patients in rural and underserved settings, HFpEF-specific RPM trials (currently underrepresented), cost-effectiveness analyses comparing RPM modalities, and longer-term follow-up studies to assess the sustainability of benefit. Given the cumulative mortality signal, future work may be more informative as comparative effectiveness and implementation research than as simple RPM-versus-no-monitoring trials.

## Conclusions

In this systematic review, meta-analysis, and trial sequential analysis of randomized controlled trials of remote patient monitoring versus usual care in adults with heart failure, remote patient monitoring significantly reduced all-cause mortality with moderate certainty of evidence, and the prediction interval excluded the null. Trial sequential analysis supported a stable mortality signal under the prespecified assumption, although publication-bias sensitivity attenuated the estimate. Heart failure hospitalization was significantly reduced with low certainty of evidence, although the prediction interval crossed the null and indicated that in some contexts, the effect may be attenuated or absent, consistent with moderate heterogeneity. No statistically significant differential effect by remote patient monitoring technology type was detected. Geographic access context remains a critical reporting gap, with the overwhelming majority of included trials not reporting rural versus urban subgroups; dedicated geographic access-enriched trials are needed to determine whether remote patient monitoring can address disparities in heart failure care delivery.

## Appendices

Appendix A: Database sensitivity assessment

Embase and CINAHL were not searched due to the absence of institutional access at the authors' affiliation (Independent Researcher, Rio de Janeiro, Brazil). This is a recognized deviation from Cochrane Handbook 6.4 recommendations, which advise routine inclusion of Embase. Database-level search strings and per-database record yields are reported in Table 1 (Materials and Methods). Five complementary lines of evidence were used to assess the likely impact of this omission.

Cochrane CENTRAL Partially Aggregates Embase Content

The Cochrane CENTRAL Register of Controlled Trials systematically incorporates randomized trial records from Embase. In the present search, 917 of the 2,138 CENTRAL records (42.9%) carried an Embase identifier, indicating that a substantial portion of Embase-indexed RCTs in the field of remote monitoring and heart failure were captured indirectly.

PubMed Combined With CENTRAL Captures the Majority of Relevant RCTs

Slobogean et al. [12] demonstrated that PubMed plus CENTRAL identifies approximately 97% of relevant RCTs in therapeutic systematic reviews. Sampson et al. [13] reported that CENTRAL captures a growing proportion of Embase-indexed RCTs over time.

Direct PubMed Indexing of Included Studies Is High

Of the 65 ultimately included RCTs, 63 (96.9%) were indexed in PubMed and would have been retrievable through PubMed alone. The remaining two records (CO-1438 (Delaney et al. (2013) [58], SAGE) and CO-0019 (Zheleznykh et al. (2025) [39], Russian-language journal)) were retrieved through CENTRAL. No included studies were Embase-only or CINAHL-only.

Cross-Referencing Against Three Contemporary 2025 Meta-Analyses With Embase Access

We compared our included study list against three independent 2025 meta-analyses that searched Embase: De Lathauwer et al. (European Journal of Heart Failure (2025); 41 RCTs; 16,312 patients) [14], Dobre et al. (American Journal of Therapeutics (2025); 105 studies; 45,072 patients) [15], and Ezimoha et al. (Cureus (2025); 15 RCTs) [16]. Mortality estimates were directionally concordant across all four reviews (RR/OR range: 0.81-0.91), and no Embase-only RCT identified by these reviews would have changed the direction or qualitative interpretation of our pooled mortality estimate.

Reflected in GRADE Assessment

The publication-bias domain in our GRADE evidence profile incorporates the residual possibility that Embase-only European or Asian RCTs were missed. Mortality certainty was downgraded one level for suspected publication bias, partly accommodating this concern.

Conclusion

The omission of Embase and CINAHL is acknowledged as a methodological limitation. Triangulation through CENTRAL overlap, PubMed indexing of included studies, and concordance with Embase-using meta-analyses supports the conclusion that the omission is unlikely to have materially altered the qualitative findings, while not eliminating residual uncertainty.

Appendix B: Screening algorithm documentation

Title and abstract screening of 2,633 unique records used a semi-automated approach with predefined exclusion rules implemented in Python (scripts/python/03_screening_ta.py, archived at the project Zenodo deposit). Records flagged by the algorithm underwent manual adjudication, with final consensus decisions retained in the screening dataset. The algorithm operated as a four-tier regex filter.

Tier 1: Animal/Non-human Exclusion

Records were excluded if the title or abstract matched animal-only research patterns (e.g., "rats," "mice," "porcine model," and "in vitro") without concomitant human terms.

Tier 2: Population Filter

Records were retained only when the title or abstract contained heart failure terms (heart failure, cardiac failure, HFrEF, HFpEF, HFmrEF, cardiomyopathy, CHF, and decompensated heart failure). The acronym "CHF" was added during a post-hoc audit (see below).

Tier 3: Intervention Filter

Records were retained only when the title or abstract contained remote monitoring terms across three categories: structured telephone support, non-invasive telemonitoring, and invasive hemodynamic monitoring. The term "telemanagement" was added during a post-hoc audit.

Tier 4: Design Filter

Records were excluded when the title contained terms indicating non-RCT design (review, editorial, commentary, case report, observational study, narrative review, and guideline). The term "guideline" alone was refined during the audit to avoid false positives.

Validation Reference Set

The algorithm was validated against eight landmark RCTs known to be eligible: GUIDE-HF, MONITOR-HF, CHAMPION, IN-TIME, BEAT-HF, TIM-HF2, TIM-HF, and Tele-HF. All eight reference trials were correctly retained by the algorithm.

Performance

Of 2,633 records screened, 1,514 were automatically excluded; 219 (8.3%) were flagged for manual adjudication, of which 116 were retained and 103 (77 + 26) were excluded; 900 records passed the algorithm directly without flagging.

Post-hoc Sensitivity Audit

A systematic audit of excluded records identified three regex gaps: missing "telemanagement" in the intervention term list, missing the standalone acronym "CHF" in the population term list, and an overly broad "guideline" pattern in the design exclusion list. Two false negatives were recovered: CO-0203 (Benatar et al. (2003) [87]) and CO-1045 (Giordano et al. (2009) [88]). One additional record (CO-0456) was flagged but excluded because the trial reported no results. The corrected algorithm achieved 96.9% sensitivity against the final 65 included studies (63 of 65 identified automatically; 2 rescued via post-hoc audit).

Limitations

The archived screening dataset retains final consensus decisions rather than complete independent reviewer-level decisions for every record. Therefore, the screening process should be described as semi-automated screening with manual adjudication and audit, rather than fully independent dual screening. Full source code, the regex term lists, and the adjudication decision log are publicly available at the project Zenodo deposit.

Appendix C: Data and code availability

All raw data, processed data, analysis code, and supplementary outputs are publicly available.

Project repository (Zenodo): https://doi.org/10.5281/zenodo.19865261

PROSPERO registration: CRD420261323628 (https://www.crd.york.ac.uk/PROSPERO/view/CRD420261323628)

medRxiv preprint: https://doi.org/10.64898/2026.02.25.26347143

The Zenodo archive contains the complete data extraction CSV with 185 variables for all 65 included trials, R analysis scripts (numbered 00-14, plus run_all.R), Python screening pipeline (01-07), risk of bias assessments, GRADE evidence profiles, sensitivity analyses, and the per-study extraction consensus log documenting reviewer roles and quality control corrections.

Fifteen post-registration protocol amendments are documented in the manuscript Methods and in the Zenodo archive.
